# Development and qualification of an enzyme-linked immunosorbent assay to detect human serum immunoglobulin G reactive to multiple lineages of Lassa virus nucleoprotein

**DOI:** 10.1371/journal.pone.0340568

**Published:** 2026-07-02

**Authors:** Heejin Yun, Faith Sigei, Nana Yaa Asiedua Appiah, Charlotte Naa Odey Quaye, Cecilia Adwoa Biaa Yankey, Eric Kyei-Baafour, Peter Hayes, Arianna Marini, Robert T. Bailer, Marija Zaric, Kwadwo Asamoah Kusi

**Affiliations:** 1 IAVI, New York, United States of America; 2 Noguchi Memorial Institute for Medical Research, University of Ghana, Accra, Ghana; Hokkaido University: Hokkaido Daigaku, JAPAN

## Abstract

Lassa fever is a severe, often fatal febrile illness endemic to West Africa caused by Lassa virus (LASV), with different virus lineages predominating across West African countries. The viral nucleoprotein (NP) is a target antigen for serological assays to identify previous exposure to LASV. To our knowledge, there is no commercially available assay that reliably quantifies anti-LASV-NP IgG antibodies in human serum. We report the development and qualification of an ELISA designed to detect and quantify anti-LASV-NP IgG in human serum samples. Following assay optimization, performance was assessed through assay qualification at clinical trial laboratories within Ghana. Assay positivity criteria, lower limit of detection, upper and lower limits of quantification, inter-assay precision, selectivity and dilutional linearity were determined. A new reference standard prepared from pooled sera from donors in endemic Lassa fever regions was established and calibrated to the first WHO international standard for LASV antibodies. One ELISA assay utilizing lineage IV LASV-NP was applicable for detection of anti-LASV-NP IgG antibodies in serum samples from different West African countries where either LASV lineages I, II, III and IV predominate. The ELISA remained selective in hemolysed serum samples with minimal loss of signal across repeated sample freeze-thaw cycles. Crucially, the developed ELISA was fully concordant with a now discontinued commercially available ELISA kit for quantification of anti-LASV-NP antibodies. Our anti-LASV-NP IgG ELISA was shown to reliably measure anti-LASV-NP IgG levels in human serum. Establishing and conducting this assay within West Africa represents an essential step towards strengthening LASV epidemiology research and supporting urgently needed development of a vaccine to prevent Lassa Fever.

## Introduction

Lassa fever (LF) is a severe, often fatal febrile illness caused by one of at least seven phylogenetic lineages of Lassa virus (LASV), a member of the *Arenaviridae* family [1–3] endemic to West Africa [4]. LASV is a zoonotic infection, the predominant host being the multimammate rat *Mastomys natalensis*, with transmission to humans via the gastrointestinal, respiratory or skin abrasion routes following contact with rodent urine or feces. Human to human transmission, including nosocomial outbreaks, may also occur via contact with blood, bodily fluids or excreta with virus excreted in urine for up to nine weeks [5]. There is currently no approved LF vaccine and LF is defined by the World Health Organization (WHO) as a public health emergency of international concern [6]. IAVI has been developing a vaccine candidate targeting LASV surface glycoprotein complex (GPC) encoded in a replication competent recombinant Vesicular Stomatitis Virus platform (rVSVΔG-LASV-GPC) [7]. The rVSVΔG platform has been successfully applied in development of a prophylactic Zaire Ebola virus vaccine rVSVΔG-ZEBOV-GP (ERVEBO®, Merck Sharp & Dohme LLC, a subsidiary of Merck & Co., Inc., Rahway, NJ, USA) that has been approved by the FDA, EMA and African countries [8–12]. rVSVΔG-LASV-GPC vaccine candidate has been tested in a Phase 1 clinical trial (IAVI C102 study: NCT04794218) [13] and is under evaluation in a Phase 2a study (IAVI C105 study: NCT05868733), across West African clinical trial sites in Liberia, Ghana and Nigeria. Such studies aim to evaluate vaccine candidate safety and immunogenicity, thereby informing on vaccine design and further evaluation in clinical trials. Vaccine candidate immunogenicity is evaluated by assessments of humoral and cellular immune responses to LASV-GPC prior to and following vaccine candidate administration.

Since LF is endemic in countries that host current and future trial centers, it is important to assess participants in clinical trials of LASV vaccine candidates for prior exposure to LASV, enabling evaluation and understanding of the vaccine candidates’ safety and immunogenicity in the context of pre-existing immune responses to LASV. Nucleoprotein (NP) is one of the five major proteins encoded by LASV [14], it is not a component of current or planned vaccine candidate regimens and can be used as a target antigen in serological assessments to identify exposure to LASV. Several lineages of LASV are endemic to West Africa with lineages I, II and III predominating in Nigeria and lineage IV in Liberia and Sierra Leone [1] with LASV vaccine development approaches to date being focused on lineage IV [5]. There is a high level of protein sequence similarity between lineages with lineage IV LASV-NP sharing 90.5%, 89.6% and 91.6% amino acid identities with lineages I, II and III LASV-NP respectively [5], with lineage IV strains spreading across West Africa originating from ancestral Nigerian strains [15,16].

Enzyme-linked immunosorbent assay (ELISA) was selected as the assay platform for serological assessment of prior LASV exposure as it enables sensitive, specific and quantitative measurement of antigen-specific IgG responses in serum for standardized assessments in clinical trial settings. Compared with alternative approaches such as rapid diagnostic tests or dried blood spot-based assays, ELISA provides higher analytical sensitivity, a broader dynamic range, and well-established compatibility with serum samples collected in vaccine trials. Importantly, ELISA allows for generation of quantitative antibody concentrations calibrated to international standards, which is essential for comparing immune responses across studies and sites.

A commercial kit detecting anti-LASV-NP antibodies in human serum (ReLASV® Pan-Lassa NP IgG/IgM ELISA Kit, Zalgen Labs, LLC) was employed in IAVI C102 clinical trial to identify trial participants with pre-existing antibody responses to LASV-NP and thereby previous exposure to LASV [13]. However, these kits are no longer available, requiring development and qualification of a new ELISA for detection of anti-LASV-NP antibodies. Development work was conducted at the Noguchi Memorial Institute for Medical Research (NMIMR), Accra, Ghana. To reduce cost and required volunteer sample volumes and increase assay throughput, ELISA development assessed the feasibility of detecting antibodies to multiple LASV-NP lineages with a single ELISA format.

The overall aim of the work was to develop and qualify an ELISA to detect anti-LASV-NP IgG antibodies in human serum, allowing assessment of participants in clinical trials of LASV vaccine candidates for potential pre and in-trial LASV exposure. The present report describes the successful development and qualification of such an assay within West Africa, which was found to be reproducible and allowed for quantification of levels of IgG specific for multiple lineages of LASV-NP with one ELISA.

## Methods

### Study approval, informed consent and inclusivity in global research

C105 clinical trial study protocol (NCT05868733) was reviewed and approved by the National Health Research Ethics Committee of Nigeria (NREC), Abuja, Nigeria; Noguchi Memorial Institute for Medical Research (NMIMR) Institutional Review Board (IRB) at the University of Ghana, Accra, Ghana and The National Research Ethics Board (NREB), Monrovia, Liberia. Additional information regarding the ethical, cultural, and scientific considerations specific to inclusivity in global research is included in supporting information (S1 File). The study groups include adults, adolescents (twelve to seventeen years of age) and children (eighteen months to five years of age and six to eleven years of age) with informed consent obtained from the parents or guardians of non-adult participants. Participants (or parents/guardians as applicable) agreeing to participate took an assessment of understanding (AOU) questionnaire. Study staff used incorrect answers on the AOU to identify aspects of the study that required clarification and focused on those areas for further review with the participant or parent/guardian. A qualified member of the study staff conducted the informed consent process by reviewing an informed consent document (ICD) and documenting it in the clinic notes. Potential participants were given adequate time to review and understand all information before agreeing to take part in the study. The participant’s or parent/guardian’s consent to participate was obtained by signing or thumb-printing. The person obtaining consent also signed and dated the consent form. The ICD was supported by simplified participant facing messaging materials such simplified information pamphlets, briefing slides and fact sheets. IAVI has developed vaccine literacy material (https://www.iavi.org/media-and-resources/vaccine-literacy-library/) which is available for use by study staff who interact directly with community members to support the informed consent process. The study started on 6^th^ March 2024 and is ongoing at the present time (May 2026).

### Blood sample collection and serum processing

Clinical trial blood samples were processed within six hours of collection. Serum tubes were centrifuged at 1,200 x*g* for 10 minutes and serum aliquots stored at −80°C.

### Enzyme-linked immunosorbent assay to detect anti-LASV-NP IgG

High-binding, flat-bottom EIA/RIA plates (Corning Incorporated, item 3590) were coated with soluble LASV-NP antigens each supplied as one production lot from Zalgen labs: lineage II (item LASV-R-0021), lineage III (item LASV-R-0031) or lineage IV (item LASV-R-0041) proteins in phosphate-buffered saline (PBS, Sigma-Aldrich, item D8537) and stored at 4°C for 16–20 hours. Initial assay development assessed optimal ELISA signal with ELISA plate wells coated with either a combination of lineage II, III and IV LASV-NP (1:1:1 ratio) or lineage IV alone at either 1 or 2 μg/mL total protein concentration, with the higher protein concentration selected for subsequent ELISA evaluation. After coating, plates were washed four times with 300 µL PBS with 0.05% v/v tween (PBS-T, Sigma-Aldrich, item P3563). Plates were blocked with 200 µL PBS with 1% w/v casein (ThermoFisher Scientific, item 37582) for 1 hour.

During assay development, a new reference pool of anti-LASV-NP IgG positive serum samples identified from C105 trial participants was generated and calibrated against a First WHO International Standard of anti-Lassa fever virus antibodies consisting of a pool of plasma samples from seven LF-convalescent individuals (NIBSC code 20/202). This WHO standard was used to generate a standard curve starting at a concentration of 50 international units (IU)/mL and followed by nine two-fold serial dilutions in PBS with 1% w/v casein and assay high (HPC) and low positive controls (LPC). An additional WHO international reference panel for anti-Lassa fever virus antibodies (NIBSC code 21/332) consisting of individual plasma samples from LF-convalescent individuals from either Nigeria (where LASV lineages I, II and III predominate) or Sierre Leone (where lineage IV predominates) was used to assess the ability of the ELISA to detect anti-LASV-NP IgG elicited by different LASV lineages. Positive controls, a negative control of a commercially supplied serum (Sigma-Aldrich, item H3667) from healthy humans not exposed to LASV, reagent blank wells (PBS 1% w/v casein) and test samples were included in triplicate in each assay plate and incubated for one hour, followed by washing as described above. HRP-labelled anti-human IgG detection antibody (Sigma, item A0170) diluted 1:10,000 was added for one hour, followed by washing as described above. Plates were developed by adding KPL Sureblue TMB 1-component peroxidase substrate (3,3,’5,5’–tetramethylbenzidine (TMB) SeraCare, item 5120–0074) for 20 minutes and the reaction stopped using TMB Stop Solution (0.5M hydrochloric acid, Microimmune, item MI20031). All incubations other than plate-coating were conducted at 37°C. Optical densities were read within five minutes using a Biotek 800TS microplate plate reader (450 nm with reference 620–650 nm). The concentration of anti-LASV-NP IgG in samples was extrapolated by four-parameter logistic (4PL) standard curves using Biotek GEN5 version 3.16 Software, Microsoft Excel and GraphPad Prism.

### Statistical analysis

Data were analyzed using GraphPad Prism version 10.6.1 (892) for Windows, GraphPad Software, San Diego, USA. A two-tailed non-parametric Wilcoxon matched pairs signed rank test assessed significant differences between two sets of paired values. A non-parametric Friedman test assessed significant differences within multiple groups of paired data. A two-tailed non-parametric Spearman test computed correlation coefficients between two variables. The threshold for significance was defined as p < 0.05. Receiver operating characteristic (ROC) curve analysis evaluated the robustness of the determined ELISA assay cutoff value.

## Results and discussion

### Initial assay establishment

**Fig 1:**
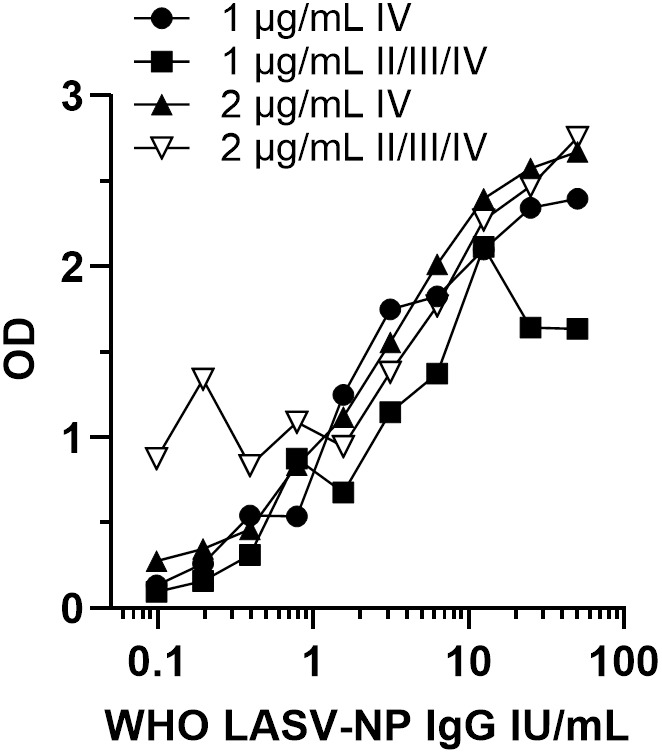
LASV-NP antigen lineage and coating concentration. A combination of LASV-NP lineage II/III/IV or lineage IV alone as coating antigens at either 1 or 2 μg/mL were assessed against a standard curve using the First WHO International Standard for anti-Lassa fever virus antibodies (NIBSC 20/202), starting at 50 IU/mL with serial two-fold dilutions. OD values are plotted against WHO-assigned international units (IU) for anti-NP antibody content of the standard. Closed circles: 1 μg/mL lineage IV, closed squares: 1 μg/mL lineages II/III/IV, closed triangles: 2 μg/mL lineage IV and open inverted triangles: 2 μg/mL lineages II/III/IV.

Initial assay development assessed optimal ELISA signal (OD_450nm_) with ELISA plate wells coated with either a combination of lineage II, III and IV (in 1:1:1 ratio) or lineage IV alone LASV-NP at 1 or 2 μg/mL total protein concentration. Fig 1 displays mean ELISA signal values plotted against WHO-assigned IU/mL for anti-NP antibody content of the First WHO International Standard for anti-Lassa fever virus antibodies (NIBSC 20/202). S1 Table displays information regarding standard curve OD and interpolated positive control values. The ELISA utilizing lineage IV LASV-NP coated at 2 µg/mL was selected for final assay qualification as this resulted in the best curve fit, an R-squared value > 0.98 at 0.996, low negative control background signal, a wide signal span across the standard curve and a positive control recovery value similar to the nominal expected value at 89% recovery (S1 Table).

### Generation of a new anti-LASV-NP IgG reference serum pool

**Fig 2:**
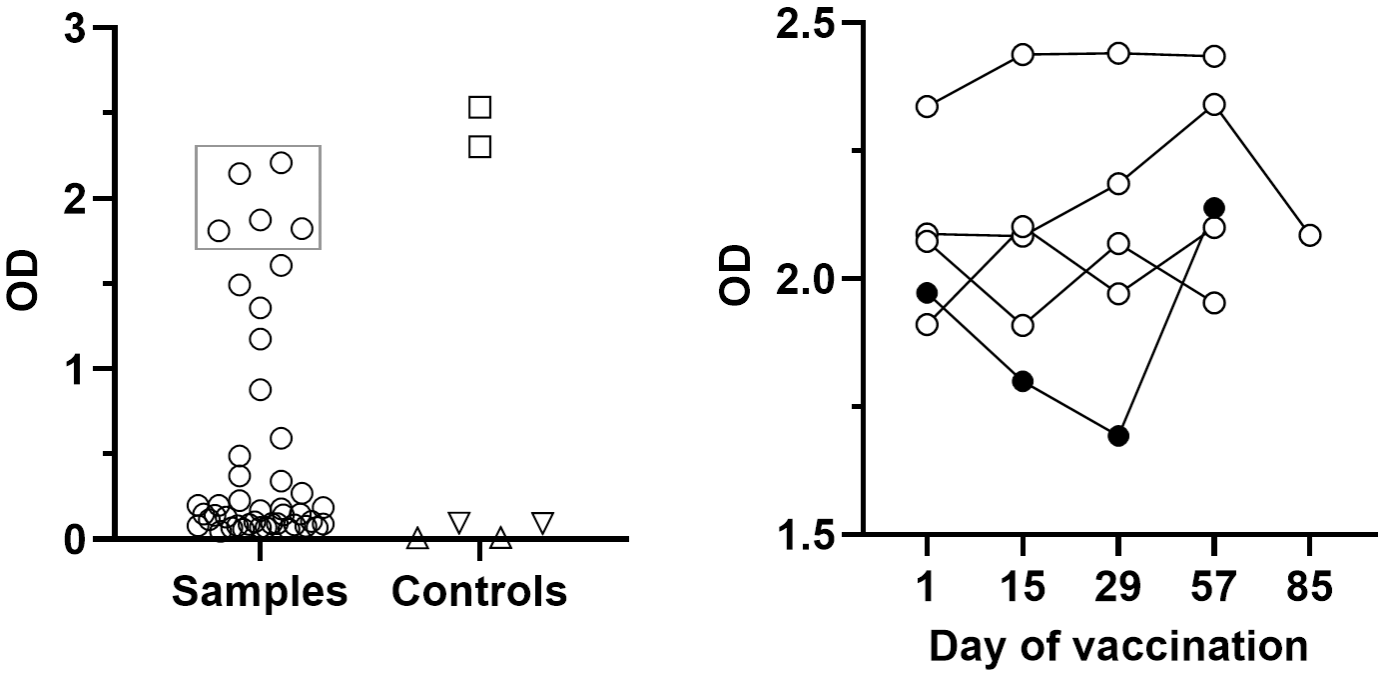
Generation of a new reference serum pool. **Left panel:** Anti-LASV-NP IgG ELISA OD values for C105 clinical trial serum samples testing positive for anti-LASV-GPC IgG prior to vaccination (circles). Additional samples from five trial participants with the highest OD values (within the box) were selected for further testing. OD values for ELISA positive controls (squares), negative controls (inverted triangles) and blank wells (triangles) are displayed. **Right panel:** Anti-LASV-NP IgG ELISA OD values for serum samples from the five selected trial participants re-tested at pre-vaccination (day 1) and post-vaccination (days 15 to 85) time points. Samples from four trial participants (open circles) were pooled to provide a new reference serum standard.

The First WHO International Standard for anti-Lassa fever virus antibodies is available in limited quantities through NIBSC (code 20/202). Therefore, a new reference standard material was produced using anti-LASV-NP IgG positive serum samples from C105 clinical trial participants for further assay development and ultimate application to clinical trial testing, with calibration against the WHO reference standard. Pre-vaccination serum samples from forty-four C105 clinical trial participants that tested positive in an ELISA [13] to detect anti-LASV-GPC IgG antibodies, were tested at 1:100 dilution in the anti-LASV-NP IgG ELISA using the conditions selected above (LASV lineage IV). Fig 2 (left panel) displays anti-LASV-NP IgG ELISA OD values for these samples and assay controls. To provide additional reference material, post-vaccination serum samples from the five participants who had the highest pre-vaccination OD values were assessed using the anti-LASV-NP IgG ELISA. All post-vaccination samples tested resulted in high OD values (Fig 2 right panel). Serum samples donated by the four trial participants with the highest OD values were pooled to provide a new reference for subsequent assay development. Therefore, all serum samples generating this new reference pool were derived from participants with serological evidence of prior LASV exposure based on the presence of both anti-LASV-NP and anti-LASV-GPC IgG antibodies. The concentration in IU/mL of this new reference serum pool was assessed in assays utilizing both LASV-NP lineages II, III and IV and IV alone as coating antigens by comparison of serially diluted new reference serum pool and WHO reference standard. At this stage of assay development, the new reference serum pool was assigned an interim concentration of 418.0 and 544.8 IU/mL for LASV-NP lineages II, III and IV and lineage IV alone, respectively (S2 Table).

### Cross lineage detection of anti-LASV-NP IgG

**Fig 3:**
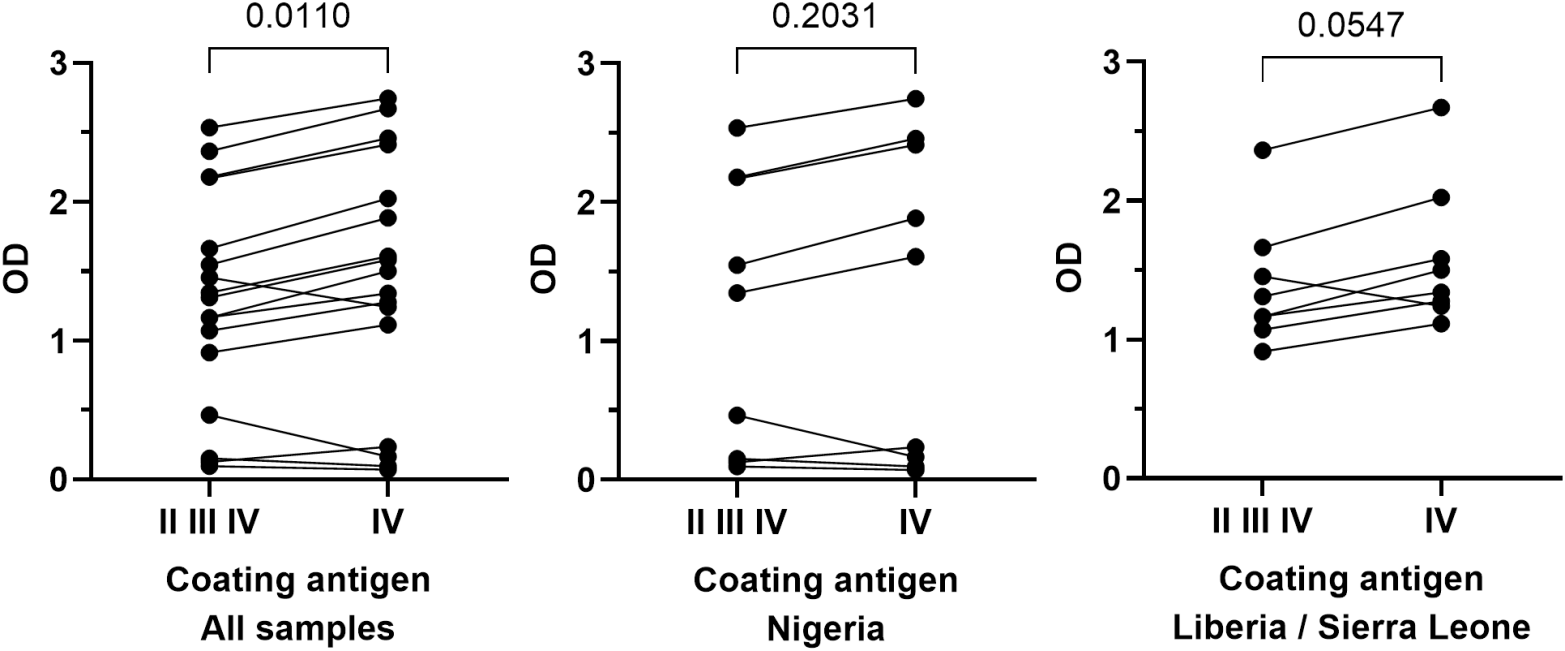
Evaluation of LASV-NP coating antigen lineages. **Left panel (all samples):** Anti-LASV-NP IgG ELISA OD values for all samples tested (WHO individual plasma sample reference panel 21/332 and C105 clinical trial serum samples testing positive for anti-LASV GPC IgG prior to vaccination) using either combined LASV-NP lineage II, III and IV or lineage IV alone as ELISA coating antigens. These data are segregated into samples from Nigeria (**middle panel**) where LASV lineages I, II and III predominate or Liberia and Sierra Leone (**right panel**) where LASV lineage IV predominates. The p values of Wilcoxon matched pairs signed rank tests between two datasets are displayed above the datapoints.

Given that LASV lineages I, II and III predominate in Nigeria, whilst lineage IV circulates in Liberia and Sierra Leone [1], it would be desirable to have one assay to detect anti-LASV-NP IgG in serum samples from all these countries. Anti-LASV-NP IgG ELISA OD signals were assessed in ten pre-vaccination anti-LASV-GPC IgG ELISA-positive C105 clinical trial samples from Nigeria and Liberia and a WHO international reference panel of seven individual LF convalescent plasma samples for anti-LASV antibodies (NIBSC 21/332) originating either from Nigeria or Sierra Leone. Two coating antigen lineage strategies were compared: LASV-NP lineage IV only and combined LASV-NP lineages II/III/IV. Fig 3 displays ELISA signal values obtained with these samples and coating antigens.

Both coating antigen lineage conditions generated ELISA signals from samples derived from all geographic locations, demonstrating that either coating antigen lineage condition can detect anti-LASV-NP IgG antibodies in samples where either LASV lineages I, II and III or lineage IV predominate. The OD signal values were marginally but significantly higher with lineage IV coating antigen compared with combined lineage II, III, and IV (median OD of 1.50 and 1.31 respectively, p = 0.0110, Wilcoxon matched pairs signed rank test, Fig 3 left panel). There was no significant difference in OD signal values between coating antigens for either Nigerian-derived (predominantly LASV lineages I, II and III) samples (median OD lineage IV and II, III and IV of 1.61 and 1.35 respectively, p = 0.2031, Fig 3 middle panel) or Liberian and Sierra Leonean-derived samples (predominantly LASV lineage IV) samples (median OD lineage IV and II, III and IV of 1.42 and 1.24 respectively, p = 0.0547, Fig 3 right panel). Coating antigen lineage IV (2 μg/mL) was chosen for further assay development as this resulted in optimal ELISA signal for samples from Nigeria, Sierra Leone and Liberia.

### Evaluation of ELISA incubation parameters

The following ELISA incubation parameters were evaluated using lineage IV LASV-NP at 2 μg/mL as coating antigen: dilution (1:5,000 or 1:10,000) of anti-human IgG detection antibody, sample (60 or 120 minutes), detection antibody (60 or 120 minutes) and TMB substrate (10 or 20 minutes) incubation times and assay incubation temperature (room temperature or 37°C). OD values of serially diluted new reference pool are displayed in S1 Fig (left panel) for the different assay parameters. Initial assessment of assay parameters indicated better signals in terms of range of OD values across the standard curves and lower negative control OD values with 20 minutes TMB substrate incubation time. ELISA parameters of detection antibody dilution, sample incubation time and assay incubation temperature were further investigated in 10 different negative control samples with the aim of minimizing background signal (S1 Fig right panel). Based on higher specific and lower background signals the optimal ELISA parameters were chosen of 60-minute sample and detection antibody incubation times with detection antibody diluted 1:10,000, 20-minute incubation of TMB substrate with the ELISA conducted at 37°C.

**Fig 4:**
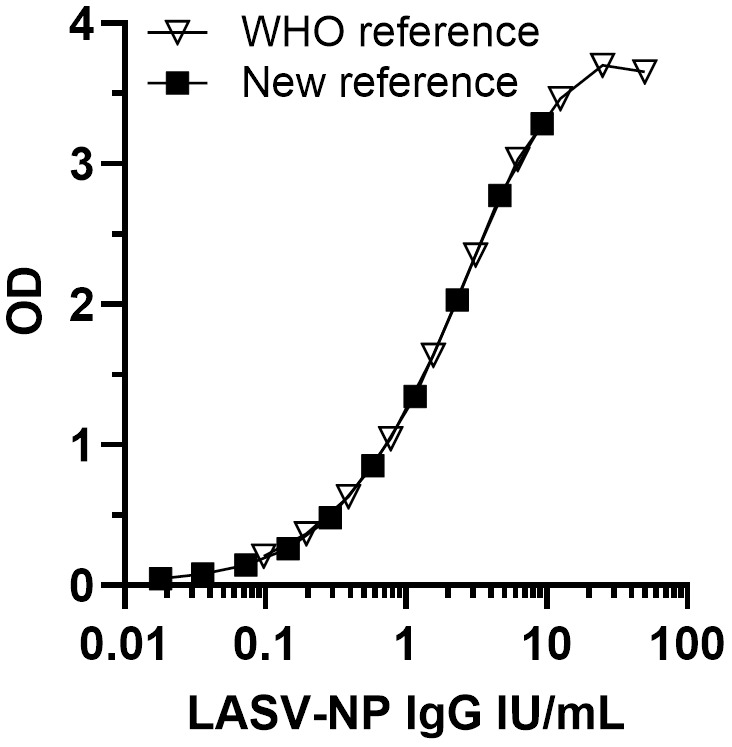
Comparison of new reference pool and WHO reference standard. Anti-LASV-NP IgG ELISA OD values for standard curves with serial two-fold dilutions of new reference pool or WHO 20/202 standard under final assay conditions. OD values are plotted against IU/mL values assigned to the new reference pool or WHO-assigned values for the WHO standard. Closed squares: new reference pool, open triangle: WHO standard.

Using the new ELISA parameters with LASV-NP lineage IV as coating antigen, the new reference serum pool was assigned a concentration of 464.6 IU/mL by comparison of serially diluted new reference serum pool and WHO 20/202 reference standard (S3 Table). ELISA OD values of serially diluted new reference pool and WHO reference standard are displayed in Fig 4.

### Assay cutoff

**Fig 5:**
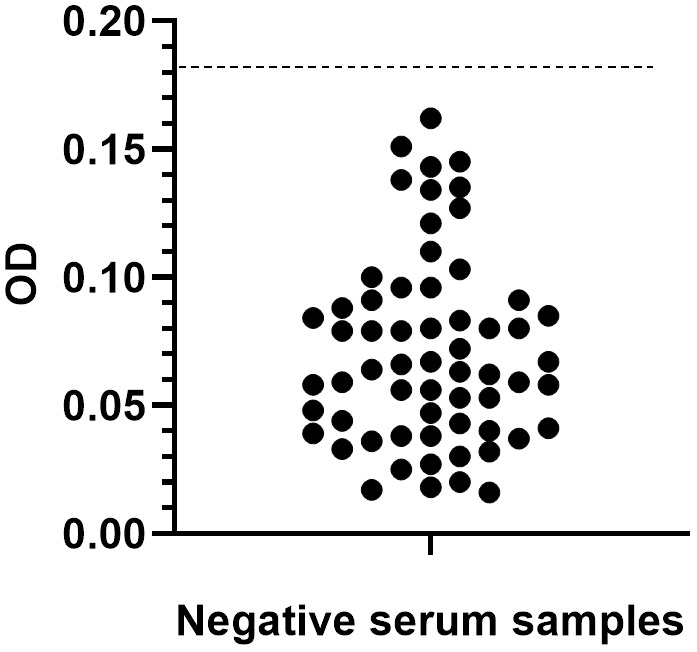
Determination of assay cutoff. Mean ELISA OD values for serum samples from sixty-one healthy Ghanaian volunteers with no history of LF. The dotted line represents the assay cutoff OD value of 0.182.

Serum samples from sixty-three healthy Ghanaian volunteers with no history of LF were tested using the LASV-NP IV lineage ELISA using the newly established assay parameters. Each sample was tested by three operators: each operator testing every sample three times. Two outlier samples with mean ODs of 2.99 and 0.226 were identified and excluded using the ROUT test (Q = 1%). OD values for the remaining sixty-one samples are displayed in Fig 5 and S4 Table. The assay cutoff was determined to be an OD of 0.182 by calculating the mean OD (0.071) of these values and adding three standard deviations (3 x 0.037). Therefore, samples yielding OD values of ≤0.182 were defined as anti-LASV-NP IgG negative in this ELISA. Based on OD values of the sixty-one samples with no history of LF and the seven samples of the WHO international reference panel for anti-Lassa fever virus antibodies (NIBSC code 21/332), ROC curve analysis determined that this cutoff value is appropriate and would correctly identify anti-LASV-NP IgG positive samples at 100% sensitivity and correctly identify negative samples at between 98.4% and 100% specificity.

### Anti-LASV-NP IgG ELISA dynamic range and positive controls

The assay upper limit of quantification (ULOQ) and lower limit of quantification (LLOQ) were defined as the highest and lowest concentrations, respectively, at which the percent recovery between measured and expected values fell within the 75–125% recovery range and remained precise with coefficients of variation (CV) <25%. ULOQ and LLOQ were assessed with a ten-point curve using a two-fold serial dilution series starting from a 1:60 dilution of the new reference serum pool in duplicate wells. The measured concentrations of each point of this curve were interpolated from a standard curve generated using two-fold serial dilutions of the reference serum pool starting at 1:50 dilution. Nine determinations were performed by three different operators, with three independent runs conducted on separate days allowing for assessment of assay precision (%CV). A low positive control (LPC) consisting of a 1:1000 dilution of the new reference serum pool was chosen to generate an anti-LASV-NP IgG value approximately four times above the LLOQ at 0.47 IU/mL. A lower dilution of 1:250 of the new reference serum pool was selected as a high positive control (HPC) below the ULOQ, equating to 1.86 IU/mL.

Data are displayed in Table 1. Dilution series ODs and interpolated IU/mL were precise (CV < 25%) over the first seven dilutions equating to an ULOQ of 7.74 IU/mL and a LLOQ of 0.12 IU/mL, with the LLOQ resulting in OD values (mean 0.187) above the assay cutoff (OD 0.182). Recovery of interpolated IU/mL values over this range was between 94.7% and 99.6% compared with nominal values. Both HPC and LPC yielded precise IU/mL values with accurate recoveries between 98.3% and 107.3% of expected values. Acceptable positive control recoveries of between 75% and 125% of expected values would equate between 0.349 to 0.581 and 1.394 to 2.323 anti-LASV-NP IgG IU/mL for LPC and HPC, respectively.

**Table 1:** ELISA dynamic range and controls.

Tested material	Mean OD	%CV OD	Nominal concentration IU/mL	Mean interpolated concentration IU/mL	%CV interpolated concentration IU/mL	% Recovery
Standard curves						
Dilution 1	2.887	11.93	7.744	7.587	11.07	98.0
Dilution 2	2.236	14.60	3.872	3.707	8.73	95.7
Dilution 3	1.566	15.40	1.936	1.872	11.39	96.7
Dilution 4	0.977	18.77	0.968	0.917	10.93	94.7
Dilution 5	0.575	17.96	0.484	0.464	9.21	95.8
Dilution 6	0.326	19.94	0.242	0.233	8.79	96.5
Dilution 7	0.187	21.92	0.121	0.120	15.71	99.6
Dilution 8	0.103	26.62	0.060	0.056	15.55	92.0
Dilution 9	0.063	35.00	0.030	0.027	25.90	88.7
Dilution 10	0.041	48.36	0.015	0.014	42.05	92.6
Controls						
HPC	1.618	21.49	1.858	1.993	22.45	107.3
LPC	0.572	25.33	0.465	0.457	20.09	98.3

Anti-LASV-NP IgG ELISA dynamic range is shown by serial dilutions of the new reference serum pool. Precision (%CV) and accuracy (% recovery) of expected values across the dilution range are indicated along with performance of high (HPC) and low positive (LPC) anti-LASV-NP IgG controls.

### Inter-assay precision and specificity

Assay precision describes the variability across repeated measures of the same test samples. Inter-assay precision was evaluated by testing twenty serum samples previously determined to be positive for anti-LASV-NP IgG responses consisting of the WHO international reference panel for anti-Lassa fever virus antibodies (NIBSC 21/332) and C105 trial pre-vaccination participants’ samples. Each sample was tested nine times; three times each by three operators on separate occasions with %CV values determined across the replicates as a measure of assay precision. IU/mL values for the expected LASV-NP IgG positive samples were precise with seventeen of the twenty (85%) samples yielding CVs of less than 25% (Table 2). Two of the three samples with CVs > 25% (C105-12 and C105-13 at 27.9% and 72.4%, respectively) were determined to have IU/mL values around the assay LLOQ (0.12 IU/mL) and therefore may be expected to yield more variable data.

**Table 2:** ELISA inter-assay precision.

	Anti-LASV-NP IgG values (IU/mL)	
Sample ID	Mean	%CV
NIBSC-20/226	12.44	9.11
NIBSC-20/244	10.11	22.35
NIBSC-20/228	8.88	11.24
NIBSC-20/204	7.87	12.11
C105-01	6.45	18.28
C105-02	6.33	10.51
C105-03	3.87	20.08
NIBSC-20/222	3.63	9.27
C105-04	3.08	21.75
C105-05	3.00	14.83
NIBSC-20/246	2.73	21.34
C105-06	2.35	33.94
C105-07	2.29	20.30
C105-08	2.03	18.74
C105-09	1.94	21.54
C105-10	1.85	10.33
NIBSC-20/248	1.77	16.45
C105-11	0.81	19.43
C105-12	0.18	72.39
C105-13	0.08	27.87

Mean anti-LASV-NP IgG ELISA IU/mL values and precision (%CV) across nine assay tests for each sample evaluated by three operators. Samples are ranked from highest to lowest mean IU/mL values. Individual NIBSC samples are part of WHO international reference panel for anti-Lassa fever virus antibodies (NIBSC code 21/332).

Assay specificity was assessed for these twenty anti-LASV-NP IgG positive samples and the sixty-one negative samples from healthy Ghanaian volunteers with no history of LF previously used to determine assay positivity criteria (Fig 5). Each data value was assigned as positive (OD > 0.182) or negative (OD ≤ 0.182) and the concordance determined between the nine tests across three operators for each sample. For the assay to be deemed to be specific, eight (88.9%) of the nine determinations for each sample must be concordant with this achieved in at least 80% of samples. This requirement was achieved for the expected negative samples with fifty-one of sixty-one (83.6%) samples being fully concordant in all nine determinations and 54 (88.5%) of these samples concordant in at least eight of nine determinations (S4 Table). All expected negative samples with concordance of less than eight of nine determinations had mean OD values considerably higher than the mean OD of the group (OD 0.071) between OD 0.121 and 0.162. Such values would be more likely to fall into either positive or negative determinations around the assay cutoff of OD 0.182. The requirement was also met for expected positive samples (S5 Table) with eighteen of twenty (90%) of samples being fully concordant. The two samples with less than eight of nine determinations being concordant yielded mean OD values of 0.12 and 0.25, close to the assay cutoff.

### Dilution linearity

**Fig 6:**
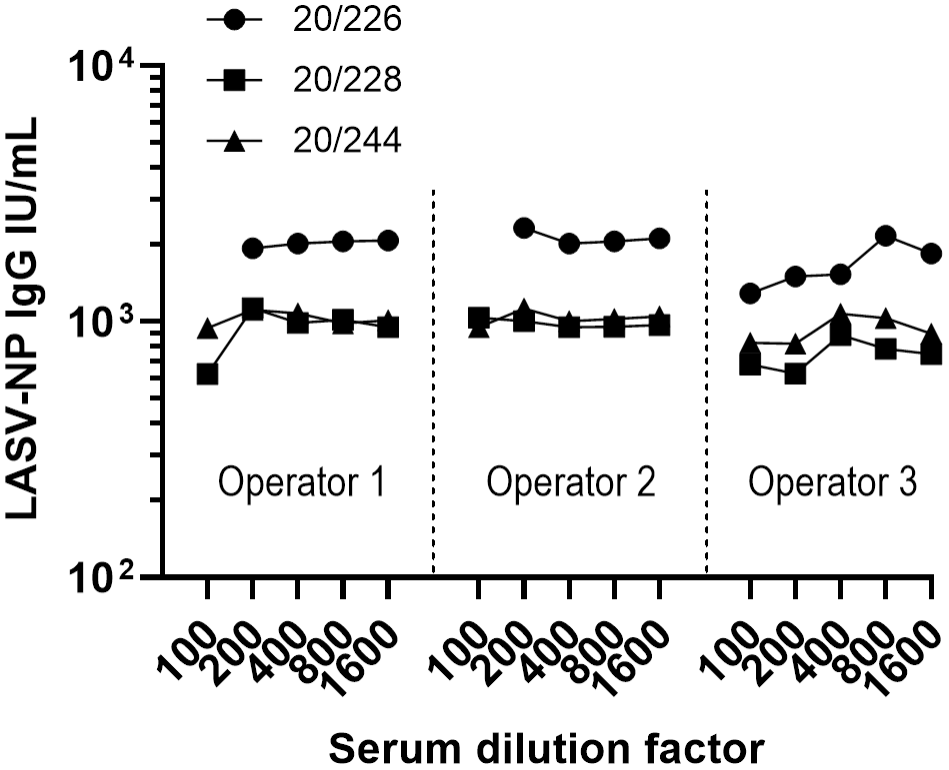
Assay dilution linearity. Dilution linearity of anti-LASV-NP IgG positive samples across dilutions from 1:100 to 1:1,600 each tested by three operators. Three of the individual WHO reference panel 21/332 samples were tested: NIBSC 20/226 (circle), NIBSC 20/228 (square) and NIBSC 20/224 (triangle) are displayed. Values for 1:100 dilutions of sample 20/226 for operators 2 and 3 were > ULOQ.

To demonstrate that assay values are not affected by sample dilution, assay dilution linearity was assessed by serially diluting three anti-LASV-NP IgG positive serum samples to span the range of the standard curve. Dilutions were tested independently by three operators and reported in IU/mL following adjustment for the dilution factor in Fig 6 and S6 Table. Assay accuracy across the 1:100–1:1,600 dilutions tested was demonstrated by linear results for anti-LASV-NP IgG concentrations (IU/mL) across these dilutions, with CVs across the three operators being less than 25%. Simple linear regression of anti-LASV-NP IgG IU/mL values prior to dilution adjustment against the reciprocal of the serum dilution factor demonstrated dilution linearity with R-squared values of at least 0.9887 for eight of the nine determinations (S6 Table).

### Concordance between commercial kit and developed ELISA

Serum samples with a range of anti-LASV-NP IgG levels determined using the developed ELISA (ten negative and eleven positive samples) were retested with both the developed ELISA and discontinued commercial ReLASV® Pan-Lassa NP IgG/IgM ELISA Kit, to evaluate concordance in assay specificity between these two methods. Each sample was tested by two operators in both assays. 100% concordance was observed for positive and negative determinations between the assays (Table 3). A direct comparison of quantitative outputs of the two assays was not possible as the commercial kit reports anti-LASV-NP IgG values in μg/mL and the developed assay in IU/mL. However, there was a strong and significant positive correlation between these positive sample values for the two assays (r = 0.964, p < 0.0001, non-parametric Spearman correlation) (S2 Fig).

**Table 3:** Concordance between commercial and developed ELISAs.

Serum Sample	Commercial ELISA kit Mean IgG μg/mL	Developed in-house ELISA Mean IgG IU/mL	Test result across all ELISAs
NS-01	NA^a^	NA^a^	Negative
NS-02	NA^a^	NA^a^	Negative
NS-03	NA^a^	NA^a^	Negative
NS-04	NA^a^	NA^a^	Negative
NS-05	NA^a^	NA^a^	Negative
NS-06	NA^a^	NA^a^	Negative
NS-07	NA^a^	NA^a^	Negative
NS-08	NA^a^	NA^a^	Negative
NS-09	NA^a^	NA^a^	Negative
NS-10	NA^a^	NA^a^	Negative
C105-14	2.90	4.75	Positive
C105-15	3.17	5.73	Positive
C105-16	1.90	3.36	Positive
C105-17	1.09	2.88	Positive
NIBSC-20/204	4.91	7.41	Positive
NIBSC-20/222	1.70	3.45	Positive
NIBSC-20/226	8.73	>ULOQ^b^	Positive
NIBSC-20/228	5.29	9.01	Positive
NIBSC-20/244	7.95	8.96	Positive
NIBSC-20/246	1.44	2.49	Positive
NIBSC-20/248	1.04	1.60	Positive

Full concordance in anti-LASV-NP IgG ELISA results between the commercial and developed ELISAs. Individual NIBSC samples are part of WHO international reference panel for anti-Lassa fever virus antibodies (NIBSC code 21/332). ^a^NA: not applicable (negative samples). ^b^ULOQ: upper limit of quantification.

### Anti-LASV-NP IgG stability over serum freeze-thaw cycles

**Fig 7:**
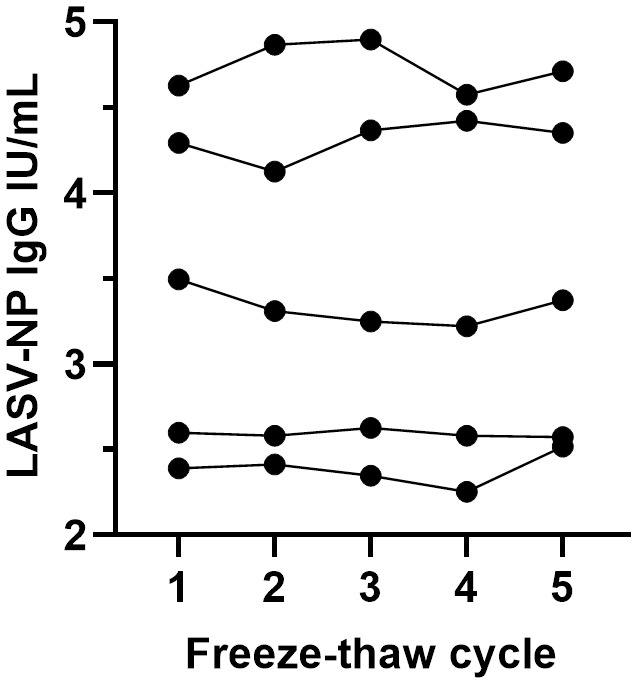
Freeze-thaw stability. Stability of anti-LASV-NP IgG IU/mL values in five anti-LASV-NP IgG positive serum samples over 1 to 5 freeze-thaw cycles between −80°C and room temperature.

Five anti-LASV-NP IgG positive serum samples were aliquoted and subjected to one to five freeze-thaw cycles (frozen at −80°C for at least one hour and thawed and held at ambient temperature for least one hour). Freeze-thaw aliquots were simultaneously assessed for anti-LASV-NP IgG IU/mL content. Fig 7 demonstrates that IU/mL values were stable with no significant differences in values over the five freeze-thaw cycles (p = 0.578, non-parametric paired Friedman test).

### Assay selectivity in hemolyzed serum samples

A small number of clinical trial serum samples may originate from whole blood samples that have undergone hemolysis, releasing red blood cell contents that may affect ELISA selectivity: the ability of the assay to detect the analyte in the presence of potentially inferring serum components. Hemolyzed human serum samples were obtained from individuals with no known exposure to LASV and shown to be negative for anti-LASV-NP IgG using the developed ELISA, with ODs between 0 and 0.140, below the assay cutoff of OD 0.182. Five anti-LASV-NP IgG positive samples were spiked into hemolyzed serum and tested on four occasions by two operators. This assay was considered selective if anti-LASV-NP IgG values for positive serum samples spiked into hemolyzed serum were between 80% and 120% of values for positive sera in the absence of hemolyzed serum. Assay selectivity in the presence of hemolyzed serum was demonstrated by the overall mean recovery of 87.3% (range 70.4% to 106.1%) of anti-LASV-NP IgG IU/mL relative to un-hemolyzed serum samples. Recoveries between 80% and 120% were achieved for all five positive samples with this achieved in seventeen out of twenty (85%) occasions across the replicate tests (Table 4).

**Table 4:** ELISA selectivity in hemolyzed serum.

LASV-NP IgG positive serum sample	% recovery of LASV-NP IgG spiked in to hemolyzed serum				
	Run 1	Run 2	Run 3	Run 4	Mean
C105-22	99.4	78.8	90.4	85.4	88.5
C105-23	95.4	84.6	81.0	86.1	86.8
C105-24	75.3	106.1	90.0	96.3	91.9
C105-25	91.1	88.8	93.9	90.8	91.1
C105-26	80.2	81.1	70.4	81.0	78.2
Mean	88.3	87.9	85.1	87.9	87.3

Per cent recovery of anti-LASV-NP IgG values for positive serum samples in the presence of hemolyzed serum compared to un-hemolyzed serum.

### ELISA assay acceptance criteria and data analysis

ELISA plate and test sample acceptance criteria were established based on the collated qualification data of this study. Table 5 displays means, standard deviations and %CVs for reagent blank and negative control OD values and LPC and HPC IU/mL values along with information regarding the standard curves. The difference between the standard curve upper (D) and lower (A) asymptote interpolated IU/mL values of the 4-PL fitting curve and R-squared values as a measure of good fit to the curve are reported. For an ELISA plate result to be accepted, the reagent blank OD must be < 0.1, negative control OD must be < 0.182 and low and high positive controls must have recoveries of 75% to 125% of expected anti-LASV-NP IgG IU/mL values (0.349 ≤ LPC ≤ 0.581 and 1.394 ≤ HPC ≤ 2.323). The standard curve must have an R-squared value ≥ 0.98 with the difference between the upper (D) and lower (A) asymptote interpolated IU/mL values (D-A) being ≥ 1.895, which equates to the mean of these values minus three standard deviations. If any of these assay acceptance criteria are not met, the whole plate must be repeated.

**Table 5:** Collated ELISA qualification data for assay reagent blank, controls and standard curves.

Statistic	Reagent blank	Negative control	Low positive control	High positive control	D^a^-A^b^	Standard curve R-squared
Number of values	90	90	90	90	90	90
Mean	0.006	0.056	0.432	1.776	4.097	0.996
Standard deviation	0.005	0.022	0.106	0.401	0.734	0.009
% CV	78.3	40.2	24.5	22.6	17.9	0.94

Means, standard deviations and %CVs across ninety evaluations of reagent blank and negative control OD values and LPC and HPC IU/mL values along with information regarding the standard curves. The difference between the standard curve upper (D) and lower (A) asymptote interpolated IU/mL values of the 4-PL fitting curve and r-squared values as a measure of good fit to the curve are reported. ^a^D: standard curve upper asymptote, ^b^A: standard curve lower asymptote.

OD values across replicate wells for individual standard curve and positive controls data points must have CVs ≤ 25%. All test samples within an ELISA plate that does not meet these criteria warrant repeat testing. Within an acceptable ELISA plate, one of three replicate wells of an individual positive test sample can be excluded if OD values have a CV > 25%. The sample would warrant repeat testing if the CV is still >25%. All test samples within one ELISA plate warrant repeat testing if more than four individual samples show CVs > 25%. Replicate wells for standard curve, negative controls and reagent blank wells cannot be excluded.

The assay dynamic range extends from 0.121 IU/mL: the LLOQ, to 7.744 IU/mL: the ULOQ. Samples producing OD values below the established cutoff of 0.182 at a 1:100 dilution are classified as negative and assigned an arbitrary concentration value of 1 IU/mL for data analysis. Samples with OD values above 0.182 are classified as positive. If a positive sample generates a signal above the cutoff but below the LLOQ at a 1:100 dilution, a final concentration of 5 IU/mL is assigned for data analysis. Samples exceeding the ULOQ (7.744 IU/mL) must be retested at a higher dilution. Final anti-LASV NP IgG concentrations should incorporate the dilution factor used for testing. Table 6 provides a summary of all assay acceptance criteria.

**Table 6:** Acceptance criteria for anti-LASV-NP IgG ELISA assay.

Parameter	Acceptance criterion	Comments
Reagent blank (OD450nm)	≤ 0.10	The ELISA plate fails if this control is > 0.1 OD
Assay cutoff (OD450nm)	≤ 0.182	Test samples with OD of ≤0.182 are deemed negative. Test samples with OD > 0.182 are deemed positive
Lower limit of quantification (LLOQ) (IgG IU/mL)	0.121	Lower limit of reliable quantification
Low Positive Control (IgG IU/mL)	0.349 ≤ LPC ≤ 0.581	The ELISA plate fails if values are outside of this range (75% to 125% recovery)
High Positive Control (IgG IU/mL)	1.394 ≤ HPC ≤ 2.323	The ELISA plate fails if values are outside of this range (75% to 125% recovery)
Upper limit of quantification (ULOQ) (IgG IU/mL)	7.744	Upper limit of reliable quantification
CV (OD450nm)	≤ 25%	Replicate wells of standard curve, controls and test samples must show CV values of ≤25%. Does not apply to negative test samples. If test sample CV > 25%, a total of four data points may be masked comprising: A single data point per triplicate for a maximum of four samples No data points may be masked for the standard curve, the negative controls, or the blank wells
Standard curve R-squared	*≥* 0.980	The ELISA plate fails if r-squared < 0.98
4PL standard curve asymptote D – asymptote A (IgG IU/mL)	≥ 1.895	The ELISA plate fails if D-A < 1.895

## Conclusions

ELISA is a vital tool for measuring antibodies against LASV-NP, enabling sensitive and specific detection of IgG responses targeting this antigen. Since LASV-NP is highly immunogenic [17–19], antibody detection provides critical information for diagnosing infection, assessing immunity and conducting sero-epidemiological studies. This assay is essential for monitoring population exposure, guiding public health interventions and supporting vaccine development against LASV. Laboratory assays, including ELISA, should be subject to rigorous qualification and standardization prior to application to clinical trials, thereby generating reliable and reproducible data allowing meaningful head-to-head comparisons of investigational products across trials. Such inter-trial comparisons of data are key to prioritizing certain vaccine candidates for further development based on both acceptable safety and most appropriate immunogenicity profiles. Previous reports describe the use of ELISA for sero-epidemiological studies of LASV-exposure by detection of LASV-GPC and LASV-NP serum antibodies [17,19–22] without details of ELISA development and qualification. As current LF vaccine candidates are primarily designed to elicit antibody responses to the virion surface glycoprotein (LASV-GPC) [23], LASV-exposure status would require assessment of LASV-NP serum antibodies in volunteers prior to, during and following participation in such clinical trials. The now discontinued Zalgen labs LASV® Pan-Lassa NP IgG/IgM ELISA Kit represented the only standardized commercial option for LASV-NP-based serology.

We report the successful development and qualification of a standardized anti-LASV-NP IgG ELISA suitable for application to clinical trials of LF vaccine candidates entirely at a clinical immunology laboratory in Ghana. This represents a key milestone for Lassa fever research in West Africa, as it demonstrates that complex immunogenicity endpoint assays can be established and performed in endemic regions, reducing reliance on distant reference laboratories. To provide sufficient material for extensive ELISA testing in clinical trials, a new serum reference pool was developed from West African pre-vaccination serum samples containing both anti-LASV-GPC and anti-LASV-NP IgG antibodies (Fig 2), a strong indication of prior LASV-exposure. The new reference pool was calibrated against the First WHO international standard for Lassa fever antibodies (Fig 4, S2 and S3 Tables) allowing for a quantitative assay output in anti-LASV-NP IgG in IU/mL, facilitating comparison of data across different studies. The developed ELISA and commercial kit were shown to have equivalent performance in qualitative detection of anti-LASV-NP IgG, with 100% concordance in samples being deemed positive or negative between assays (Table 3). There was a strong positive correlation in quantification of anti-LASV-NP IgG values (r = 0.964, p < 0.0001, non-parametric Spearman correlation, S2 Fig), although with different reported units of IgG concentration (IU/mL and μg/mL, respectively).

The developed ELISA utilizing LASV-NP lineage IV as coating antigen allowed detection of IgG reactive to NP from the different LASV lineages predominating across West Africa. There were no significant differences in ELISA signals when either LASV-NPs of lineages II / III / IV or IV alone were used as coating antigens for samples originating from Nigeria, where lineage I, II and III predominate or Liberia and Sierra Leone where lineage IV predominates (Fig 3), presumably due to the relatively high level of shared amino acid sequence between LASV-NP lineages [5]. Screening of clinical trial samples to detect serum IgG reactivity to NP from multiple LASV lineages is facilitated in terms of cost, time and sample volumes with one ELISA utilizing lineage IV NP as coating antigen. Assay qualification demonstrated a reproducible assay with acceptable sensitivity, specificity, selectivity and precision. The assay was shown to be reliable and reproducible when performed by different operators, with precise low %CV values that were accurate to expected IgG standard concentrations over the assay dynamic range of 0.12 to 7.74 IU/mL (Table 1). Clear discrimination in ELISA signal was demonstrated between serum samples from subjects previously exposed to LASV (Table 2) and those with no exposure history (S4 Table and Fig 5). Linear concentrations of IgG were determined over a typical serum sample assay dilution range of 1:100–1:1600 (Fig 6 and S6 Table). Stable anti-LASV-NP IgG concentrations were detected over repeated freeze-thaw cycles (Fig 7) which would allow for repeat sample testing as required without loss of signal. Assay robustness to variations in sample quality that may be encountered in the clinic was demonstrated with acceptable selectivity maintained in hemolysed sera samples with mean recovery of 87.3% of anti-LASV-NP IgG IU/mL in hemolysed serum samples compared with unhemolysed samples (Table 4).

This study has several limitations. Firstly, assay performance was evaluated using samples from West African clinical trial cohorts and further validation in other populations would strengthen generalizability. Secondly, while cross-lineage reactivity was demonstrated, the number of samples representing each lineage was limited. Thirdly, only one production lot of LASV-NP antigens was available for testing from the supplier, testing of additional lots would strengthen considerations of assay robustness. Finally, potential cross-reactivity with other arenaviruses was not evaluated and warrants further investigation.

Creation of a new LASV reference serum pool calibrated to the WHO International Standard enabling quantitative reporting in IU/mL, ensures sustainability for future clinical trials and sero-epidemiological studies. The reference serum pool can be made available to the scientific community upon reasonable request, subject to appropriate material transfer agreements and ethical considerations. This ELISA provides a standardized, reproducible and multi-lineage compatible tool for assessing anti-LASV-NP IgG responses and monitoring vaccine-induced or natural immunity. By developing and establishing this capability locally, the study enhances the ability of West African laboratories to conduct high-quality immunological assessments in populations most affected by Lassa fever and other pathogens.

## Supporting information

S1 Fig Optimization of assay conditions. Left panel: ELISA OD values under different assay conditions. Detection antibody dilutions are indicated as 1:5,000 (closed data points) and 1:10,000 (open data points). Assay parameters of time of sample, detection antibody and TMB substrate incubation and incubation temperature are respectively indicated by circles: 120 minutes, 60 minutes, 10 minutes, room temperature, squares: 120 minutes, 60 minutes, 10 minutes, 37°C, triangles: 120 minutes, 60 minutes, 20 minutes, room temperature, inverted triangles: 60 minutes, 60 minutes, 20 minutes, 37°C and diamonds: 60 minutes, 30 minutes, 10 minutes, 37°C. Right panel: ELISA OD values for 10 LASV negative serum samples tested with either detection antibody diluted at 1:5,000 or 1:10,000 and incubated at room temperature (RT) or 37°C. Ab: antibody.
(TIF)

S2 Fig Correlation between commercial and developed ELISAs. Correlation between quantification of anti-LASV-NP IgG values determined in serum samples using a commercial Zalgen ELISA kit (expressed in μg/mL) and the developed ELISA (expressed in IU/mL). Spearman correlation demonstrates a strong and significant positive correlation between the two assays (r = 0.964, p < 0.0001).
(TIF)

S1 Table Standard curve OD values and positive control concentrations. (DOCX)

S2 Table Derivation of new reference pool concentration under interim assay conditions. (DOCX)

S3 Table Derivation of new reference pool concentration under final assay conditions. (DOCX)

S4 Table Assay specificity with negative samples. (DOCX)

S5 Table Assay specificity with positive samples. (DOCX)

S6 Table ELISA dilution linearity. (DOCX)

S1 File Inclusivity in global research. (DOCX)

S1 Data Supporting information Raw Data. (XLSX)
