# Supplementary figures and images for: Development and qualification of an enzyme-linked immunosorbent assay to detect human serum immunoglobulin G reactive to multiple lineages of Lassa virus nucleoprotein
Source: PLoS One. 2026 Jul 2;21(7):e0340568. doi: 10.1371/journal.pone.0340568 (PMC13327249; doi:10.1371/journal.pone.0340568)

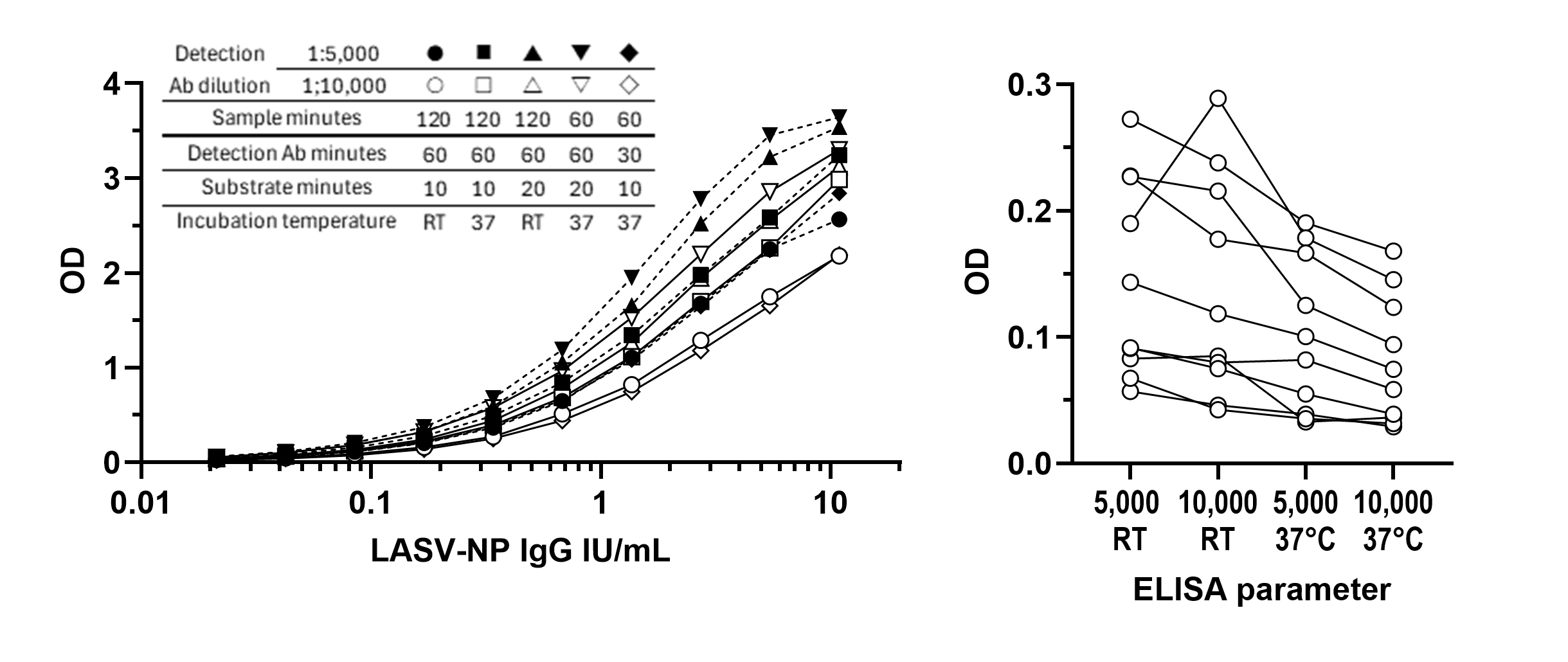

Supplement: S1 Fig — Left panel: ELISA OD values under different assay conditions. Detection antibody dilutions are indicated as 1:5,000 (closed data points) and 1:10,000 (open data points). Assay parameters of time of sample, detection antibody and TMB substrate incubation and incubation temperature are respectively indicated by circles: 120 minutes, 60 minutes, 10 minutes, room temperature, squares: 120 minutes, 60 minutes, 10 minutes, 37°C, triangles: 120 minutes, 60 minutes, 20 minutes, room temperature, inverted triangles: 60 minutes, 60 minutes, 20 minutes, 37°C and diamonds: 60 minutes, 30 minutes, 10 minutes, 37°C. Right panel: ELISA OD values for 10 LASV negative serum samples tested with either detection antibody diluted at 1:5,000 or 1:10,000 and incubated at room temperature (RT) or 37°C. Ab: antibody. (TIF) [file pone.0340568.s001.tif]

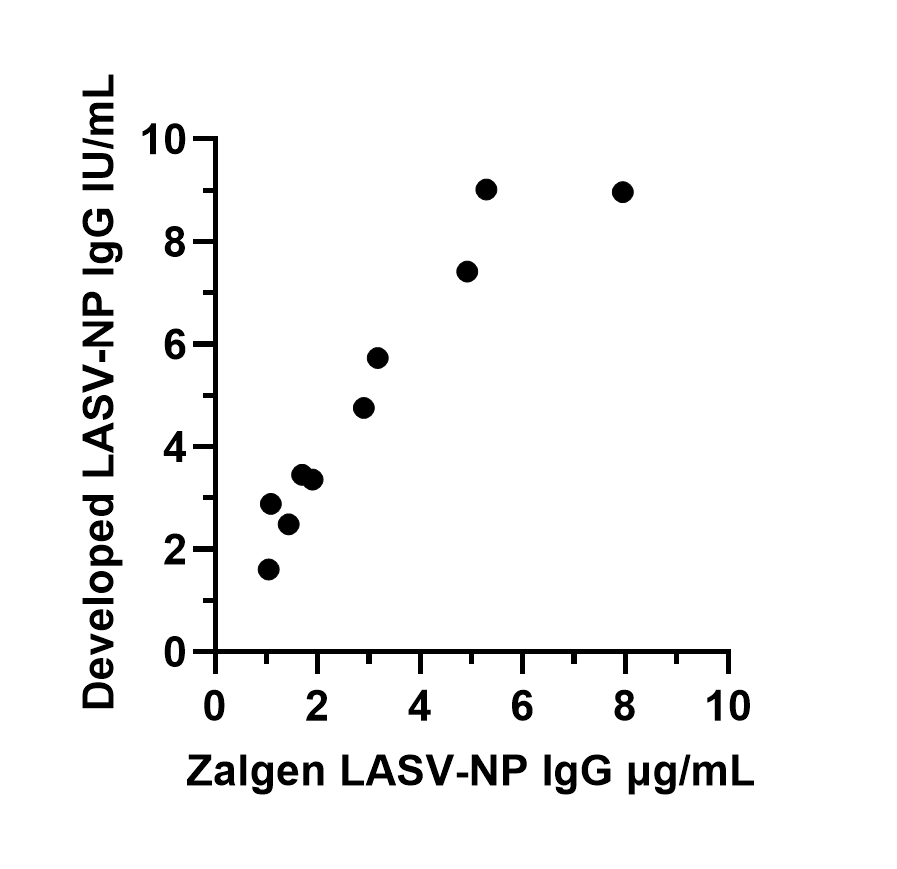

Supplement: S2 Fig — Correlation between quantification of anti-LASV-NP IgG values determined in serum samples using a commercial Zalgen ELISA kit (expressed in μg/mL) and the developed ELISA (expressed in IU/mL). Spearman correlation demonstrates a strong and significant positive correlation between the two assays (r = 0.964, p < 0.0001). (TIF) [file pone.0340568.s002.tif]
