# Supplementary material for: Development and qualification of an enzyme-linked immunosorbent assay to detect human serum immunoglobulin G reactive to multiple lineages of Lassa virus nucleoprotein
Source: PLoS One. 2026 Jul 2;21(7):e0340568. doi: 10.1371/journal.pone.0340568 (PMC13327249; doi:10.1371/journal.pone.0340568)
Supplement: S1 Table — (DOCX) [file pone.0340568.s003.docx]

**S1 Table.** **Standard curve OD values and positive control concentrations**

|  | LASV-NP coating concentration (total protein) and lineage | | | |
| --- | --- | --- | --- | --- |
| Nonlinear Curve Fit | 1 µg/mL  IV | 1 µg/mL  II/III/IV | 2 µg/mL  IV | 2 µg/mL  II/III/IV |
| Bottom | 0.050 | 0.040 | 0.180 | 0.999 |
| Top | 2.417 | 1.843 | 2.830 | 2.744 |
| IC50 | 1.732 | 1.727 | 2.760 | 7.273 |
| Hill Slope | 1.096 | 1.075 | 1.006 | 1.762 |
| LogIC50 | 0.239 | 0.237 | 0.441 | 0.862 |
| Span | 2.367 | 1.803 | 2.650 | 1.744 |
| R squared | 0.932 | 0.814 | 0.996 | 0.763 |
| Interpolated IU/mL concentration of Positive Control (Nominal concentration: 10 IU/mL) | 4.303 | 6.207 | 8.901 | 9.220 |
| Negative Control (OD) | 0.201 | 0.058 | 0.099 | 0.098 |
