# Supplementary material for: Development and qualification of an enzyme-linked immunosorbent assay to detect human serum immunoglobulin G reactive to multiple lineages of Lassa virus nucleoprotein
Source: PLoS One. 2026 Jul 2;21(7):e0340568. doi: 10.1371/journal.pone.0340568 (PMC13327249; doi:10.1371/journal.pone.0340568)
Supplement: S2 Table — (DOCX) [file pone.0340568.s004.docx]

**S2 Table: Derivation of new reference pool concentration under interim assay conditions**

|  | |  | **Lineage II/III/IV**  **Mean = 417.992 IU/mL** | | | | **Lineage IV**  **Mean = 544.847 IU/mL** | | | |
| --- | --- | --- | --- | --- | --- | --- | --- | --- | --- | --- |
|  | |  | Mean | 434.425 | Mean | 401.560 | Mean | 550.496 | Mean | 539.197 |
|  | |  | SD | 55.507 | SD | 22.205 | SD | 29.064 | SD | 22.920 |
|  | |  | CV% | 12.777 | CV% | 5.530 | CV% | 5.280 | CV% | 4.251 |
| Serum pool and standard curve dilutions | | | Run 1 | | Run 2 | | Run 1 | | Run 2 | |
| Dilution Factor | Pool / STD | | Interpolated Concentration | Result | Interpolated Concentration | Result | Interpolated Concentration | Result | Interpolated Concentration | Result |
| 50 | Pool STD1 | | 10.02 | 500.999 | 7.382 | 369.116 | 12.138 | 606.876 | 10.441 | 522.042 |
| 100 | Pool STD2 | | 4.783 | 478.306 | 4.181 | 418.067 | 5.479 | 547.882 | 5.169 | 516.897 |
| 200 | Pool STD3 | | 2.359 | 471.718 | 2.029 | 405.841 | 2.671 | 534.219 | 2.803 | 560.665 |
| 400 | Pool STD4 | | 1.001 | 400.263 | 1.033 | 413.215 | 1.323 | 529.145 | 1.393 | 557.184 |
| 800 | Pool STD5 | | 0.478 | 382.042 | 0.497 | 397.828 | 0.666 | 532.757 | 0.706 | 565.124 |
| 1600 | Pool STD6 | | 0.233 | 373.221 | 0.251 | 401.795 | 0.345 | 552.099 | 0.347 | 555.942 |
| 3200 | Pool STD7 | | 0.120 | 385.590 | 0.108 | 345.757 | 0.175 | 561.060 | 0.156 | 497.680 |
| 6400 | Pool STD8 | | 0.066 | 420.072 | 0.045 | 288.777 | 0.089 | 567.761 | 0.064 | 409.402 |
| 12800 | Pool STD9 | | 0.040 | 513.184 | 0.015 | 190.232 | 0.047 | 600.475 | 0.009 | 118.547 |
| 25600 | Pool STD10 | | 0.029 | 734.687 | NA^a^ | NA^a^ | 0.027 | 701.632 | NA^a^ | NA^a^ |

Determination of interim anti-LASV-NP IgG concentrations (IU/mL) in a new reference serum pool by serial dilution of pool and WHO reference standard (NIBSC 20/202). The new reference serum pool was assigned values of 418.0 and 544.8 IU/mL for LASV-NP lineages II, III and IV and lineage IV alone, respectively. ^a^NA: not available.
