# Supplementary material for: Development and qualification of an enzyme-linked immunosorbent assay to detect human serum immunoglobulin G reactive to multiple lineages of Lassa virus nucleoprotein
Source: PLoS One. 2026 Jul 2;21(7):e0340568. doi: 10.1371/journal.pone.0340568 (PMC13327249; doi:10.1371/journal.pone.0340568)
Supplement: S3 Table — (DOCX) [file pone.0340568.s005.docx]

**S3 Table. Derivation of new reference pool concentration under final assay conditions**

|  | |  | **Lineage IV**  **Mean = 464.6 IU/mL** | | | | | |
| --- | --- | --- | --- | --- | --- | --- | --- | --- |
|  | |  | Mean | 465.350 | Mean | 493.514 | Mean | 434.983 |
|  | |  | SD | 19.753 | SD | 27.346 | SD | 6.996 |
|  | |  | CV% | 4.245 | CV% | 5.541 | CV% | 1.608 |
| Serum pool and standard curve dilutions | | | Run 1 | | Run 2 | | Run 3 | |
| Dilution Factor | Pool / STD | | Interpolated Concentration | Result | Interpolated Concentration | Result | Interpolated Concentration | Result |
| 50 | Pool STD1 | | 9.425 | 471.227 | 9.696 | 484.805 | 8.611 | 430.526 |
| 100 | Pool STD2 | | 4.621 | 462.144 | 4.589 | 458.906 | 4.281 | 428.065 |
| 200 | Pool STD3 | | 2.213 | 442.597 | 2.424 | 484.808 | 2.190 | 437.984 |
| 400 | Pool STD4 | | 1.139 | 455.433 | 1.269 | 507.513 | 1.108 | 443.355 |
| 800 | Pool STD5 | | 0.619 | 495.347 | 0.664 | 531.538 | 0.534 | 427.128 |
| 1600 | Pool STD6 | | 0.296 | 473.901 | 0.329 | 526.682 | 0.278 | 444.447 |
| 3200 | Pool STD7 | | 0.110 | 351.855 | 0.139 | 443.304 | 0.124 | 396.289 |
| 6400 | Pool STD8 | | NA^a^ | NA^a^ | 0.010 | 66.420 | 0.054 | 346.633 |
| 12800 | Pool STD9 | | NA^a^ | NA^a^ | NA^a^ | NA^a^ | 0.021 | 262.763 |
| 25600 | Pool STD10 | | NA^a^ | NA^a^ | NA^a^ | NA^a^ | 0.004 | 113.088 |

Determination of final anti-LASV-NP IgG concentration (IU/mL) in a new reference serum pool by serial dilution of pool and WHO reference standard (NIBSC 20/202). The new reference serum pool was assigned a value of 464.6 IU/mL. ^a^NA: not available.
