# Supplementary material for: Development and qualification of an enzyme-linked immunosorbent assay to detect human serum immunoglobulin G reactive to multiple lineages of Lassa virus nucleoprotein
Source: PLoS One. 2026 Jul 2;21(7):e0340568. doi: 10.1371/journal.pone.0340568 (PMC13327249; doi:10.1371/journal.pone.0340568)
Supplement: S4 Table — (DOCX) [file pone.0340568.s006.docx]

**S4 Table. Assay specificity with negative samples**

|  | Operator 1 OD values | | | Operator 2 OD values | | | Operator 3 OD values | | | All operators | | |
| --- | --- | --- | --- | --- | --- | --- | --- | --- | --- | --- | --- | --- |
| Serum  sample | RUN 1 | RUN 2 | RUN 3 | RUN 1 | RUN 2 | RUN 3 | RUN 1 | RUN 2 | RUN 3 | Mean | %CV | % results negative  OD ≤0.182 |
| NS-11 | 0.124 | 0.082 | 0.116 | 0.118 | 0.075 | 0.071 | 0.106 | 0.069 | 0.062 | 0.091 | 26.7 | 100 |
| NS-12 | 0.122 | 0.105 | 0.141 | 0.077 | 0.070 | 0.067 | 0.093 | 0.082 | 0.061 | 0.091 | 29.7 | 100 |
| NS-13 | 0.097 | 0.075 | 0.075 | 0.054 | 0.046 | 0.046 | 0.059 | 0.045 | 0.033 | 0.059 | 33.8 | 100 |
| NS-14 | 0.169 | 0.140 | 0.123 | 0.100 | 0.055 | 0.058 | 0.119 | 0.057 | 0.046 | 0.096 | 46.0 | 100 |
| NS-15 | 0.131 | 0.207 | 0.223 | 0.133 | 0.115 | 0.122 | 0.125 | 0.115 | 0.129 | 0.145 | 28.1 | 77.8 |
| NS-16 | 0.091 | 0.075 | 0.061 | 0.095 | 0.045 | 0.047 | 0.071 | 0.045 | 0.031 | 0.062 | 35.7 | 100 |
| NS-17 | 0.115 | 0.077 | 0.077 | 0.080 | 0.043 | 0.048 | 0.062 | 0.044 | 0.033 | 0.064 | 39.8 | 100 |
| NS-18 | 0.117 | 0.022 | 0.071 | 0.087 | 0.049 | 0.057 | 0.072 | 0.051 | 0.042 | 0.063 | 44.0 | 100 |
| NS-19 | 0.000 | 0.157 | 0.145 | 0.119 | 0.081 | 0.070 | 0.125 | 0.091 | 0.075 | 0.096 | 49.6 | 100 |
| NS-20 | 0.146 | 0.100 | 0.132 | 0.094 | 0.082 | 0.076 | 0.128 | 0.071 | 0.066 | 0.100 | 29.4 | 100 |
| NS-21 | 0.145 | 0.186 | 0.163 | 0.155 | 0.114 | 0.123 | 0.144 | 0.110 | 0.100 | 0.138 | 20.4 | 88.9 |
| NS-22 | 0.203 | 0.132 | 0.188 | 0.144 | 0.101 | 0.132 | 0.144 | 0.092 | 0.081 | 0.135 | 30.5 | 77.8 |
| NS-23 | 0.240 | 0.050 | 0.057 | 0.049 | 0.026 | 0.051 | 0.054 | 0.030 | 0.039 | 0.066 | 99.5 | 100 |
| NS-24 | 0.026 | 0.025 | 0.018 | 0.018 | 0.010 | 0.012 | 0.023 | 0.012 | 0.003 | 0.016 | 48.0 | 100 |
| NS-25 | 0.136 | 0.087 | 0.098 | 0.067 | 0.047 | 0.049 | 0.103 | 0.066 | 0.060 | 0.079 | 36.9 | 100 |
| NS-26 | 0.115 | 0.135 | 0.102 | 0.071 | 0.062 | 0.061 | 0.088 | 0.072 | 0.052 | 0.084 | 33.2 | 100 |
| NS-27 | 0.030 | 0.029 | 0.025 | 0.013 | 0.012 | 0.012 | 0.020 | 0.017 | 0.003 | 0.018 | 51.0 | 100 |
| NS-28 | 0.039 | 0.031 | 0.027 | 0.024 | 0.020 | 0.020 | 0.032 | 0.018 | 0.009 | 0.025 | 35.6 | 100 |
| NS-29 | 0.166 | 0.140 | 0.124 | 0.116 | 0.083 | 0.081 | 0.132 | 0.078 | 0.068 | 0.110 | 30.6 | 100 |
| NS-30 | 0.061 | 0.035 | 0.049 | 0.035 | 0.025 | 0.041 | 0.037 | 0.025 | 0.034 | 0.038 | 30.3 | 100 |
| NS-31 | 0.070 | 0.059 | 0.053 | 0.052 | 0.034 | 0.049 | 0.053 | 0.033 | 0.027 | 0.048 | 29.2 | 100 |
| NS-32 | 0.074 | 0.076 | 0.069 | 0.057 | 0.031 | 0.048 | 0.051 | 0.063 | 0.050 | 0.058 | 25.2 | 100 |
| NS-33 | 0.041 | 0.042 | 0.067 | 0.042 | 0.019 | 0.032 | 0.038 | 0.038 | 0.037 | 0.040 | 31.6 | 100 |
| NS-34 | 0.151 | 0.152 | 0.187 | 0.189 | 0.080 | 0.148 | 0.113 | 0.087 | 0.101 | 0.134 | 30.5 | 77.8 |
| NS-35 | 0.102 | 0.094 | 0.105 | 0.097 | 0.045 | 0.074 | 0.067 | 0.063 | 0.071 | 0.080 | 25.9 | 100 |
| NS-36 | 0.051 | 0.087 | 0.098 | 0.040 | 0.019 | 0.046 | 0.037 | 0.036 | 0.060 | 0.053 | 47.8 | 100 |
| NS-37 | 0.044 | 0.048 | 0.056 | 0.047 | 0.020 | 0.031 | 0.025 | 0.032 | 0.031 | 0.037 | 32.3 | 100 |
| NS-38 | 0.115 | 0.100 | 0.143 | 0.073 | 0.037 | 0.065 | 0.051 | 0.063 | 0.066 | 0.079 | 42.5 | 100 |
| NS-39 | 0.073 | 0.087 | 0.086 | 0.067 | 0.045 | 0.066 | 0.057 | 0.066 | 0.056 | 0.067 | 20.4 | 100 |
| NS-40 | 0.044 | 0.055 | 0.046 | 0.037 | 0.022 | 0.027 | 0.025 | 0.023 | 0.021 | 0.033 | 37.5 | 100 |
| NS-41 | 0.052 | 0.049 | 0.057 | 0.034 | 0.022 | 0.024 | 0.026 | 0.030 | 0.031 | 0.036 | 36.0 | 100 |
| NS-42 | 0.039 | 0.029 | 0.059 | 0.026 | 0.014 | 0.019 | 0.018 | 0.019 | 0.018 | 0.027 | 53.3 | 100 |
| NS-43 | 0.025 | 0.043 | 0.050 | 0.030 | 0.020 | 0.024 | 0.026 | 0.032 | 0.043 | 0.032 | 32.2 | 100 |
| NS-44 | 0.043 | 0.072 | 0.080 | 0.060 | 0.028 | 0.032 | 0.040 | 0.103 | 0.048 | 0.056 | 44.0 | 100 |
| NS-45 | 0.189 | 0.138 | 0.212 | 0.106 | 0.047 | 0.098 | 0.112 | 0.068 | 0.114 | 0.121 | 43.8 | 77.8 |
| NS-46 | 0.065 | 0.044 | 0.055 | 0.043 | 0.021 | 0.038 | 0.038 | 0.039 | 0.049 | 0.044 | 28.4 | 100 |
| NS-47 | 0.228 | 0.228 | 0.237 | 0.184 | 0.069 | 0.157 | 0.124 | 0.094 | 0.137 | 0.162 | 37.9 | 55.6 |
| NS-48 | 0.180 | 0.172 | 0.186 | 0.146 | 0.074 | 0.149 | 0.112 | 0.127 | 0.138 | 0.143 | 24.9 | 88.9 |
| NS-49 | 0.087 | 0.078 | 0.099 | 0.065 | 0.012 | 0.048 | 0.042 | 0.039 | 0.060 | 0.059 | 45.6 | 100 |
| NS-50 | 0.055 | 0.052 | 0.060 | 0.043 | 0.020 | 0.040 | 0.029 | 0.029 | 0.039 | 0.041 | 32.2 | 100 |
| NS-51 | 0.094 | 0.145 | 0.138 | 0.073 | 0.028 | 0.080 | 0.055 | 0.056 | 0.076 | 0.083 | 46.2 | 100 |
| NS-52 | 0.175 | 0.096 | 0.155 | 0.080 | 0.063 | 0.054 | 0.043 | 0.049 | 0.078 | 0.088 | 53.1 | 100 |
| NS-53 | 0.084 | 0.084 | 0.072 | 0.055 | 0.045 | 0.036 | 0.035 | 0.035 | 0.054 | 0.056 | 36.1 | 100 |
| NS-54 | 0.023 | 0.022 | 0.024 | 0.014 | 0.009 | 0.011 | 0.025 | 0.010 | 0.013 | 0.017 | 38.9 | 100 |
| NS-55 | 0.062 | 0.060 | 0.064 | 0.036 | 0.030 | 0.032 | 0.034 | 0.025 | 0.040 | 0.043 | 35.7 | 100 |
| NS-56 | 0.079 | 0.088 | 0.082 | 0.052 | 0.059 | 0.038 | 0.043 | 0.041 | 0.044 | 0.058 | 33.4 | 100 |
| NS-57 | 0.062 | 0.056 | 0.099 | 0.036 | 0.025 | 0.052 | 0.027 | 0.026 | 0.037 | 0.047 | 51.3 | 100 |
| NS-58 | 0.263 | 0.206 | 0.285 | 0.136 | 0.126 | 0.095 | 0.020 | 0.087 | 0.145 | 0.151 | 56.6 | 66.7 |
| NS-59 | 0.127 | 0.101 | 0.110 | 0.062 | 0.067 | 0.056 | 0.023 | 0.046 | 0.060 | 0.072 | 46.2 | 100 |
| NS-60 | 0.132 | 0.117 | 0.139 | 0.068 | 0.059 | 0.055 | 0.036 | 0.046 | 0.071 | 0.080 | 48.1 | 100 |
| NS-61 | 0.055 | 0.052 | 0.071 | 0.032 | 0.022 | 0.026 | 0.047 | 0.021 | 0.026 | 0.039 | 45.7 | 100 |
| NS-62 | 0.055 | 0.052 | 0.047 | 0.030 | 0.022 | 0.026 | 0.057 | 0.023 | 0.027 | 0.038 | 39.2 | 100 |
| NS-63 | 0.043 | 0.042 | 0.047 | 0.020 | 0.020 | 0.014 | 0.050 | 0.014 | 0.018 | 0.030 | 51.6 | 100 |
| NS-64 | 0.036 | 0.022 | 0.038 | 0.015 | 0.013 | 0.020 | 0.007 | 0.014 | 0.017 | 0.020 | 52.1 | 100 |
| NS-65 | 0.138 | 0.134 | 0.202 | 0.103 | 0.078 | 0.088 | 0.029 | 0.055 | 0.097 | 0.103 | 49.5 | 88.9 |
| NS-66 | 0.127 | 0.123 | 0.136 | 0.078 | 0.076 | 0.063 | 0.034 | 0.053 | 0.075 | 0.085 | 41.8 | 100 |
| NS-67 | 0.122 | 0.100 | 0.153 | 0.082 | 0.072 | 0.073 | 0.006 | 0.050 | 0.063 | 0.080 | 52.6 | 100 |
| NS-68 | 0.093 | 0.086 | 0.073 | 0.045 | 0.035 | 0.043 | 0.010 | 0.041 | 0.048 | 0.053 | 50.0 | 100 |
| NS-69 | 0.124 | 0.095 | 0.114 | 0.067 | 0.058 | 0.063 | 0.037 | 0.071 | 0.080 | 0.079 | 35.3 | 100 |
| NS-70 | 0.133 | 0.108 | 0.089 | 0.054 | 0.048 | 0.046 | 0.019 | 0.035 | 0.069 | 0.067 | 55.0 | 100 |
| NS-71 | 0.206 | 0.199 | 0.197 | 0.134 | 0.115 | 0.098 | 0.018 | 0.073 | 0.104 | 0.127 | 50.3 | 66.7 |

Assay specificity demonstrated by anti-LASV-NP IgG ELISA OD values for sixty-one negative samples across nine tests performed by three operators. Negative values are defined as having an OD of ≤0.182. For the assay to be deemed to be specific, at least eight (88.9%) of the nine determinations for each sample must be concordant with this achieved in at least 80% of samples. In this case, determinations for fifty-four of sixty-one (88.5%) samples were concordant.
