# Supplementary material for: Development and qualification of an enzyme-linked immunosorbent assay to detect human serum immunoglobulin G reactive to multiple lineages of Lassa virus nucleoprotein
Source: PLoS One. 2026 Jul 2;21(7):e0340568. doi: 10.1371/journal.pone.0340568 (PMC13327249; doi:10.1371/journal.pone.0340568)
Supplement: S5 Table — (DOCX) [file pone.0340568.s007.docx]

**S5 Table. Assay specificity with positive samples**

|  | Operator 1 OD values | | | Operator 2 OD values | | | Operator 3 OD values | | | All operators | | |
| --- | --- | --- | --- | --- | --- | --- | --- | --- | --- | --- | --- | --- |
| Serum sample | RUN 1 | RUN 2 | RUN 3 | RUN 1 | RUN 2 | RUN 3 | RUN 1 | RUN 2 | RUN 3 | Mean OD | % CV | % results positive  OD >0.182 |
| NIBSC-20/226 | 3.47 | 3.40 | 2.57 | 3.52 | 3.30 | 3.12 | 3.55 | 2.78 | 3.48 | 3.24 | 10.82 | 100 |
| NIBSC-20/244 | 2.98 | 3.34 | 2.65 | 3.05 | 3.14 | 2.91 | 3.30 | 2.32 | 3.40 | 3.01 | 11.68 | 100 |
| NIBSC-20/228 | 3.04 | 3.00 | 2.42 | 3.08 | 3.15 | 2.76 | 3.21 | 2.38 | 3.11 | 2.91 | 10.76 | 100 |
| NIBSC-20/204 | 3.04 | 2.88 | 2.30 | 3.19 | 3.06 | 2.57 | 3.09 | 2.12 | 3.04 | 2.81 | 13.74 | 100 |
| C105-27 | 2.90 | 2.66 | 2.03 | 2.93 | 2.78 | 2.52 | 2.96 | 1.97 | 2.98 | 2.64 | 14.81 | 100 |
| C105-28 | 2.79 | 2.84 | 2.16 | 2.72 | 2.98 | 2.45 | 3.04 | 1.74 | 2.98 | 2.63 | 16.64 | 100 |
| C105-29 | 2.28 | 2.52 | 1.71 | 2.26 | 2.63 | 1.86 | 2.57 | 1.37 | 2.45 | 2.18 | 20.09 | 100 |
| NIBSC-20/222 | 2.47 | 2.28 | 1.68 | 2.37 | 2.32 | 1.75 | 2.44 | 1.65 | 2.24 | 2.13 | 15.94 | 100 |
| C105-30 | 2.09 | 1.98 | 1.60 | 2.28 | 2.36 | 1.83 | 2.31 | 1.24 | 2.04 | 1.97 | 18.54 | 100 |
| C105-31 | 2.10 | 2.27 | 1.39 | 1.88 | 2.18 | 1.59 | 2.41 | 1.54 | 2.19 | 1.95 | 18.66 | 100 |
| NIBSC-20/246 | 1.90 | 1.98 | 1.39 | 1.89 | 2.14 | 1.60 | 2.49 | 1.34 | 1.98 | 1.86 | 19.63 | 100 |
| C105-32 | 1.95 | 1.81 | 1.21 | 1.50 | 1.96 | 1.46 | 2.56 | 0.98 | 1.93 | 1.71 | 27.74 | 100 |
| C105-33 | 1.78 | 1.88 | 1.24 | 1.63 | 1.95 | 1.63 | 2.15 | 1.06 | 1.97 | 1.70 | 20.92 | 100 |
| C105-34 | 1.73 | 1.91 | 1.33 | 1.54 | 1.78 | 1.43 | 1.82 | 1.02 | 1.70 | 1.58 | 17.94 | 100 |
| C105-35 | 1.81 | 1.92 | 1.05 | 1.35 | 1.94 | 1.34 | 1.95 | 0.87 | 1.77 | 1.56 | 26.52 | 100 |
| C105-36 | 1.78 | 1.73 | 0.93 | 1.50 | 1.80 | 1.25 | 1.77 | 1.09 | 1.83 | 1.52 | 22.83 | 100 |
| NIBSC-20/248 | 1.58 | 1.42 | 1.09 | 1.41 | 1.79 | 1.33 | 1.74 | 1.05 | ND | 1.43 | 18.99 | 100 |
| C105-37 | 1.01 | 0.73 | 0.58 | 0.95 | 1.11 | 0.70 | 1.24 | 0.62 | 0.91 | 0.87 | 26.29 | 100 |
| C105-38 | 0.26 | 0.64 | 0.12 | 0.13 | 0.29 | 0.15 | 0.21 | 0.14 | 0.26 | 0.25 | 65.75 | 55.6 |
| C105-39 | 0.19 | 0.11 | 0.08 | 0.07 | 0.19 | 0.08 | 0.13 | 0.07 | 0.16 | 0.12 | 41.96 | 22.2 |

Assay specificity demonstrated by anti-LASV-NP IgG ELISA OD values for twenty positive samples across nine tests performed by three operators. Positive values are defined as having an OD of >0.182. For the assay to be deemed to be specific, at least eight (88.9%) of the nine determinations for each sample must be concordant with this achieved in at least 80% of samples. In this case, determinations in eighteen of twenty (90%) samples were concordant. Individual NIBSC samples are part of WHO international reference panel for anti-Lassa fever virus antibodies (NIBSC code 21/332).
