# Supplementary material for: Development and qualification of an enzyme-linked immunosorbent assay to detect human serum immunoglobulin G reactive to multiple lineages of Lassa virus nucleoprotein
Source: PLoS One. 2026 Jul 2;21(7):e0340568. doi: 10.1371/journal.pone.0340568 (PMC13327249; doi:10.1371/journal.pone.0340568)
Supplement: S6 Table — (DOCX) [file pone.0340568.s008.docx]

**S6 Table. ELISA dilution linearity**

|  | IU/mL values over 1:100 to 1:1,600 dilution series | | | | | | | | | | |
| --- | --- | --- | --- | --- | --- | --- | --- | --- | --- | --- | --- |
|  | Operator 1 | | | Operator 2 | | | Operator 3 | | | All values | |
| Sample | Mean | R-squared | %CV | Mean | R-squared | %CV | Mean | R-squared | %CV | Mean | %CV |
| NIBSC 20/226 | 2020.8 | 0.9995 | 3.2 | 2128.1 | 0.9951 | 6.3 | 1664 | 0.9926 | 20.5 | 1916.6 | 15.6 |
| NIBSC-20/228 | 942 | 0.8433 | 20.2 | 983.4 | 0.9996 | 3.6 | 743.8 | 0.9887 | 13.4 | 889.7 | 17.9 |
| NIBSC-20/244 | 1024.9 | 0.9905 | 6.7 | 1030.2 | 0.9905 | 6.2 | 929.4 | 0.9934 | 12.7 | 994.8 | 9.4 |

Mean, R-squared and precision (%CV) values of anti-LASV-NP IgG IU/mL determinations across serial dilutions of anti-LASV-NP IgG positive samples tested by three operators. Individual NIBSC samples are part of WHO international reference panel for anti-Lassa fever virus antibodies (NIBSC code 21/332).
