# Supplementary material for: Development and qualification of an enzyme-linked immunosorbent assay to detect human serum immunoglobulin G reactive to multiple lineages of Lassa virus nucleoprotein
Source: PLoS One. 2026 Jul 2;21(7):e0340568. doi: 10.1371/journal.pone.0340568 (PMC13327249; doi:10.1371/journal.pone.0340568)
Supplement: S1 File — (DOCX) [file pone.0340568.s009.docx]

Inclusivity in global research

PLOS’ policy on inclusivity in global research aims to improve transparency in the reporting of research performed outside of researchers’ own country or community and ensures that PLOS publications reporting global research adhere to high standards for research ethics and authorship. Authors of relevant research articles may be asked to complete the questionnaire below, which outlines ethical, cultural, and scientific considerations specific to inclusivity in global research. This questionnaire may be requested when researchers have travelled to a different country to conduct research, if research uses samples collected in another country, research with Indigenous populations or their lands, or if research is on cultural artefacts. Researchers travelling to another country solely to use laboratory equipment will not normally be required to complete the questionnaire. However, the questionnaire can be requested at the journal’s discretion for any submission – if you have been requested to complete this questionnaire by the PLOS journal you submitted to, please do so.

Please complete the questionnaire below and include this as a Supporting Information file with your manuscript. Note that if your paper is accepted for publication, this checklist will be published with your article in the supporting information files. Please ensure that you reference the checklist in the main body of your manuscript. We suggest adding a subsection ‘Inclusivity in global research’ to your Methods section and adding the following sentence: “Additional information regarding the ethical, cultural, and scientific considerations specific to inclusivity in global research is included in the Supporting Information (SX Checklist)”

The questions have been designed to be applicable to a wide range of study types, and there are subsections for both human subjects research and non-human subjects research. If any of the questions are not relevant to your research please mark them as “N/A” as appropriate.

**Ethical considerations, permits and authorship**

*This section is applicable to all research types.*

Provide details as to who granted permissions and/or consent for the study to take place in the Methods section of your manuscript. This should include the names of **all** ethics boards, governmental organizations, community leaders or other bodies that provided approval for the study. If individuals provided approval refer to these people by their role or title but do not list their name(s).

Reported on page number: 6 (of the unmarked version “Manuscript” without track changes)

If there were any deviations from the study protocol after approval was obtained please provide details of these changes in the Methods section of your manuscript.

Reported on page number: N/A

Did this study involve local collaborators that are residents of the country where the research was conducted or members of the community studied? If you do not have any authors from said communities, please provide an explanation for this below.

Yes. The authorship includes local collaborators where the laboratory work for the manuscript was conducted.

Everyone listed as an author should meet PLOS’ criteria for authorship and all individuals who meet these criteria should be included in the author byline, rather than the acknowledgements. For further information please see the journal’s Authorship Policy.

**Human subjects research (e.g. health research, medical research, cross-cultural psychology)**

Did you obtain written informed consent from a representative of the local community or region before the research took place? How did you establish who speaks for the community? Details of written informed consent obtained from study participants should be reported separately in the Methods section of your manuscript.

The C105 clinical trial study protocol (NCT05868733) was reviewed and approved by the local institutional review boards at clinical trial centres in Nigeria, Liberia and Ghana. Written consent for the study was provided by each review board.

A full list of local institutional review boards is provided in the manuscript method section along with details of the procedure for obtaining written informed consent from study participants.

The study has adopted the principles of “Good Participatory Practice (GPP) for Emerging Infectious diseases”. Each site has developed GPP plans for community engagement and volunteer recruitment informed by the needs of this trial and experiences from previous clinical trials. Prior to the study initiation, community mapping was conducted to identify relevant stakeholders and gatekeepers at each of the trial sites. None of the sites require written informed consent from the community for the research to take place. However, each site has constituted a community advisory board (CAB) that represents the various stakeholders. These CABs meet at regular intervals to receive and provide feedback related to the study progress. Community liaison officers at the research site are a vital link between the community members and the research team and serve to guide the conduct of the trial in a manner that is sensitive to community norms.

How did members of the local community provide input on the aims of the research investigation, its methodology, and its anticipated outcome(s)?

All Principal investigators and research staff were given the opportunity to review and provide feedback on the overall study (objectives and methodology) prior to finalization. Lay community members mainly provided feedback about study procedures through the community advisory boards and focus group discussions with community engagement research teams.

When engaging with the local community, how did you ensure that the informed consent documents and other materials could be understood by local stakeholders?

Participants (or parents/guardians as applicable) agreeing to participate took an assessment of understanding (AOU) questionnaire. Study staff used incorrect answers on the AOU to identify aspects of the study that required clarification and focused on those areas for further review with the participant or parent/guardian.

A qualified member of the study staff conducted the informed consent process by reviewing the informed consent document (ICD) and documenting it in the clinic notes. Potential participants were given adequate time to review and understand all information before agreeing to take part in the study. The participant’s or parent/guardian’s consent to participate was obtained by him/her signing or thumb-printing. The person obtaining consent also signed and dated the consent form. The signed and/or marked and dated ICD remained at the study site. A copy of the signed/marked and dated ICD was offered to the participant or parent/guardian to take home. Those participants or parents/guardians who did not wish to take a copy were required to document that they declined to do so. If the participant or parent/guardian was functionally illiterate, the completed ICD was read to him/her in the language that he/she best understood in the presence of an independent literate observer not affiliated with the study, who signed and dated the ICD as an impartial witness.

The informed consent document is also supported by simplified participant facing messaging materials such simplified information pamphlets, briefing slides and fact sheets. IAVI has developed vaccine literacy material (<https://www.iavi.org/media-and-resources/vaccine-literacy-library/>) which is available for use by study staff who interact directly with community members to support the informed consent process.

Will the findings of the research be made available in an understandable format to stakeholders in the community where the study was conducted (e.g. via a presentation, summary report, copies of publications, etc.)? Please provide details of how this will be achieved.

Communication plans are developed at different stages of the trial. At the end of the trial, IAVI will work with principal investigators, community liaison officers at the sites and CABs to identify the key stakeholders for whom results of the trial need to be communicated. A “Results dissemination plan” will be developed and the communication format will be tailored to the audience. Copies of publications will be made available to the investigators for onward transmission to government authorities and local members of the scientific community. IAVI will support the presentation of study outcomes at local, regional and continental scientific gatherings. Simplified presentations, information pamphlets or musical jingles will be developed for lay community members in a language vetted and approved by the investigators and CABs.

**Non-human subjects research using specimens/ animals collected as part of the study, or those housed in archival collections. Examples include archaeology, paleontology, botany and zoology.**

Did the permission you obtained from a local authority to perform the study include an agreement on access to outputs and benefit sharing? This may include procedures to enable fair distribution of the benefits and resources arising from the research performed. Please include any details of Prior Informed Consent and Benefit Sharing Agreements obtained. These may be required by field-specific regulations, for example the Convention on Biological Diversity (CBD) and the associated Nagoya Protocol.

N/A

If the material used in your study was imported, please A) provide the year it was imported and B) indicate whether permits were obtained to import/export the materials used, C) provide details of any permits obtained. If this information is not available, please indicate this.

N/A

If you used archival specimens, please state how the material used in your study was acquired by the institute it is held in and provide details of any permits obtained for the original excavations/ sample collection. If this information is not available, please indicate this.

N/A

How was the potential cultural significance of the materials collected in your study to local communities considered in your research design? Were Indigenous peoples and/or local researchers and institutions involved with archaeological excavations / collection of specimens? If so, please provide a description of their involvement.

N/A

If your manuscript includes photographs of human remains please indicate whether authors obtained permission from descendants or affiliated cultural communities to do so.

N/A
