# Supplementary material for: Development and qualification of an enzyme-linked immunosorbent assay to detect human serum immunoglobulin G reactive to multiple lineages of Lassa virus nucleoprotein
Source: PLoS One. 2026 Jul 2;21(7):e0340568. doi: 10.1371/journal.pone.0340568 (PMC13327249; doi:10.1371/journal.pone.0340568)
Supplement: Attachment — Submitted filename: Response to reviewers.pdf [file pone.0340568.s014.pdf]

Dr. Peter Hayes
Senior Scientist
phayes@iavi.org
+44 (0) 7989086636

13th May 2026

Development and qualification of an enzyme-linked immunosorbent assay to detect human serum immunoglobulin G reactive to multiple lineages of Lassa virus nucleoprotein

Original submission manuscript number: PONE-D-25-67874

Dear Sir or Madam,

Thank you for inviting a resubmission of the above titled manuscript for consideration for publication in Plos One. For resubmission, I welcome and have responded to and addressed these very constructive comments both from the Editor and Reviewers as detailed below.

Response to Editor comments

1. The manuscript meets PLOS ONE's style requirements.

2. A completed copy of PLOS' questionnaire on inclusivity in global research has been uploaded as supporting information in the resubmission. As requested in this questionnaire, this checklist is referred to in the revised manuscript methods section "Study approval, informed consent and inclusivity in global research" along with the suggested sentence added at line 104 of the revised manuscript: "Additional information regarding the ethical, cultural, and scientific considerations specific to inclusivity in global research is included in the Supporting Information (S1 Checklist)".

3. The funders had no role in study design, data collection and analysis, decision to publish, or preparation of the manuscript.

4. The funding-related text has been removed from the manuscript.

5. A raw data excel file has been uploaded as Supporting information. This file contains raw data values that were used to derive means, standard deviations and other measures reported in the figures and tables in the submission.

6. Supplementary figures file type has been amended to “supporting information” in the resubmission.

7. The revised ethics statement is in methods section “study approval, informed consent and inclusivity in global research”. The full names of IRBs are included and additional information on informed consent is added at revised line 101 as suggested in the “inclusivity in global research questionnaire”.

8. Reviewers did not ask for citing of specific previously published works.

Responses to Reviewers’ comments

We thank both reviewers for the excellent contribution to this manuscript with extensive, detailed and very constructive comments of a positive nature. We very much appreciate the effort this must have entailed and believe that by addressing and incorporating reviewers’ suggestions, the revised manuscript is very much improved.

A) Responses to Reviewer #1 comments

Response to overall comments

The results section has been revised to focus on the key highlights of the study. The main body of the manuscript results section has been revised in regard to testing related to minute operator details in assay optimization, thereby allowing more focus on other findings, by: i) removal of individual operator data from tables (original tables 2 and 4, now revised tables 2 and 3) which now focus on key data of means and %CV, ii) moving original figure 4 showing effect of different incubation parameters from the main text to supporting information S1 Fig, iii) moving original table 3 for dilution linearity from the main text to supporting information S6 table and iv) removal of the section regarding manual and automated plate washing (text and original figure 7 and original table 5) as advised in reviewer #1 specific line comments below. Data tables showing reproducibility and precision across operators have been retained when they appear in the less prominent location of supporting information (S4 to S6 Tables), as reviewer #2 states precision data are one of the strengths of this work.

The discussion section has been fleshed out to provide more context as to where the data are presented in figures and tables.
The abstract has been reworked to reflect these changes to the manuscript, focusing on the key findings of the study as advised.

Response to specific line comments
Original line 68 (revised line 85): changed to “Zalgen Labs, LLC” and referred to as “Zalgen Labs” thereafter.

Original lines 61-66 and 80-88 have been combined into one paragraph (revised lines 64-75) with original lines 67-73 starting a new paragraph at revised line 84.

Original lines 73-79 (principal of the assay) have been deleted as we agree this is described in the methods and replaced with reasons for choosing ELISA to detect LASV exposure.

Original line 112 has been moved as suggested to revised line 133 in methods.

Original lines 116-118 have been removed with washing described in revised line 137 referenced “as described above” in revised lines 154-155.

Original lines 118-119: “initially” has been removed with the incubation temperature statement now at revised line 158.

Original line 130: The stop solution consists of 0.5 M hydrochloric acid (HCl), as specified by the manufacturer. This information has now been added at revised line 158.

Original lines 132-134 have been removed.

Original lines 149-155 have been removed. The starting concentration of the WHO standard curve and serial dilutions are now stated in the ELISA method section at revised line 145.

Original lines 158-160: Extrapolation of concentration has been moved to the end of the paragraph describing the ELISA method in the methods section at revised line 160.

Original line 179: The methods section from revised line 141 onwards now describes both WHO standards used for greater clarity earlier in the manuscript. The WHO control used for initial assay development and calibration with the new reference pool we generated is 20/202,

one pool of plasma reference material. Revised line 147 describes another WHO reference (21/332) consisting of seven individual plasma samples used for the cross-lineage studies (Fig 3) and as additional positive samples in revised tables 2, 3, S5 and S6.

The original manuscript S2 Table displayed derivation of new reference serum pool concentration under interim assay conditions by comparison with WHO standard 20/202. For completeness, an additional supporting information S3 Table has been added showing derivation of the concentration of the new reference serum pool under the final assay conditions, again by comparison with WHO standard 20/202. A new figure 4 has been added to the main document comparing side by side the NIBSC 20/202 and the new reference pool at the selected 2ug/mL lineage IV. Additional text describing this figure and a figure legend are added at the end of results section “Evaluation of ELISA incubation parameters”, revised line 279.

Original line 210: As discussed above, NIBSC 20/202 is one pool of convalescent plasma samples. Reference panel NIBSC 21/332 consists of seven individual LF convalescent plasma samples from Nigeria or Sierra Leone allowing for the cross-lineage studies described. The original text at line 210 (now revised line 233) has been revised to clearly state 21/332 are individual samples from Nigeria or Sierra Leone and the original text at line 123 in ELISA method now describes both the 20/202 (revised line 141) and 21/332 (revised line 147) reference materials for greater clarity.

Original line 212: The description of LASV-NP lineage ELISA plate coating has been removed in this results section and is described within the ELISA method section at revised line 130.

Original paragraph 225-238 (now revised lines 248-260) has been reworded away from emphasizing trending significance to stating there were no significant differences between NP coating antigen lineage and ability to detect LASV-NP IgG of different lineages.

To provide more focus on key points in the results as advised, the section evaluating ELISA incubation parameters (revised line 262 onwards) has been shortened by moving original figure 4 to supporting information (now S1 Fig). Similarly, original Table 3 for dilution linearity has been moved to supporting information S6 Table. The dilution linearity Fig 6 has been retained as reviewer #2 states that evaluation of assay performance including linearity are notable strengths of the manuscript, noting that reviewer #1 stated these sections “can be removed” rather than should/must be removed. The results section for comparison of manual and automatic plate washing has been removed, along with original figure 7 and table 5, as

with hindsight, authors accept that this aspect is not required, it is not specific to the ELISA method presently reported and would apply to any ELISA method.

In addition, as suggested by reviewer #1 previously, the results section is now less bloated with “minute operator details”, by removal of induvial operator data from original tables 2 and 4 in the main text (revised tables 2 and 3), now focusing on the key data with only overall means and %CV values retained.

Original line 473 and S5 Table: Supporting information S5 Table has been moved to the main text as table 6 (referred to at revised line 458 with table at revised line 475).

Original lines 494-497: The conclusions section has been revised to cite which figures or tables provide evidence for conclusions stated. The level of qualitative concordance between the two assays is now stated (100%) from revised line 506 with reference to table 3. The strong correlation in LASV-NP IgG values between the assays is now stated with reference to S2 Fig in the text.

Original lines 511-512: Text has been added at revised line 541 “The reference serum pool can be made available to the scientific community upon reasonable request, subject to appropriate material transfer agreements and ethical considerations”.

Discussion in general: Other reports describe the use of LASV-NP and LASV-GPC ELISAs for sero-epidemiologic studies in West Africa but with little or no details on ELISA development, qualification or standardization. Such reports are now referenced [17, 19-22](revised line 488). The conclusions section now discusses the need for assays applied to clinical trials to be qualified and standardized, allowing comparison of data across trials (revised line 483). We also now state (revised lines 493-498) that the present report concerns development qualification and standardization of a LASV-NP IgG ELISA to replace the discontinued Zalgen labs kit, which previously was the only standardized and commercial kit available.

The importance of development if this assay within West Africa is now stated at the end of both the abstract (revised line 36) and conclusions sections (revised line 544).

Several limitations of the study have now been added to the discussion from revised line 532 onwards.

Original line 524, Simpson has been corrected (revised line 556).

S4 Table (now S5 Table): WHO reference materials NIBSC 20/202 (one plasma pool) and NIBSC 21/332 (seven separate plasma samples) have been addressed in response to the earlier point above (original line 210). The methods section now clearly describes these two reference materials and their application (over revised lines 141-151). The footnotes to revised tables 2, 3, S5 and S6, where these individual NIBSC samples are used, also clarifies this point.

Original line 609 a commercial Zalgen ELISA kit has been amended (revised line 639).

The 2nd and 3rd abstract paragraphs have been rewritten as advised. Text describing the ELISA method and use of controls has been removed. The Abstract emphasizes work was conducted in Ghana / West Africa (revised line 24), a new reference pool calibrated to the WHO standard was generated (revised line 27), one ELISA can be used for different LASV lineages (revised line 29), hemolysed (revised line 31) and freeze/thawed samples (revised line 32) can be tested and that the developed and commercial ELISAs were concordant (revised line 32).

B) Response to reviewer #2 comments

1. Serum samples used to generate the new reference pool were selected based on LASV-NP reactivity by screening of 44 pre-vaccination C105 trial participants that tested positive in an ELISA for anti-LASV-GPC IgG antibodies, as stated in the previously submitted manuscript from original line 182 (revised line 202). Therefore, the new reference pool was generated from participants' sera that were reactive for both LASV-NP and LASV-GPC prior to any LASV vaccination. This is more clearly stated with text inserted at revised line 210 and in conclusions revised line 500.

2. Only one production lot of NP antigen was available and evaluated from the supplier and this is now stated at original line 111 (revised line 131). This point has been included as a limitation of the study in the discussion section at revised line 535.

3. As suggested, ROC curve analysis has been conducted using Graphpad prism software using the WHO seven sample reference panel (NIBSC 31/332) and the 61 naïve controls. Use of this test is stated in the statistical analysis methods section revised line 170. The analysis output from this software strengthens the cutoff of 0.182 OD based on +3 standard deviations, as this cutoff value has a sensitivity of 100% and in terms of

specificity, lies between 98.36% and 100%. The output of this analysis is described at the end of the results section "assay cutoff" at revised line 298.

4. We agree that the pandemic potential is overstated and have removed text to this effect at original line 43. We have changed the text at revised line 50 to state that the WHO defines LF as a "public health emergency of international concern". The original WHO document referenced as [6] has been replaced by another WHO document more applicable for this revised statement.

5. Although IAVI originally was an acronym for the "International AIDS Vaccine Initiative" I have been advised by the IAVI administration team that this is no longer the case, the organization's name is IAVI and this is no longer an acronym. IAVI rebranding has evolved to reflect expanded areas of activities in addition to that of HIV-1, as exemplified by the present manuscript.

6. The term "positivity point" has been replaced by "cutoff" throughout the manuscript.

7. Original line 509 has been changed to LASV at revised line 527.

As suggested, I have used PLOS's NAAS figure tool. Figures and supporting figures were uploaded to the webtool which stated "Image complies with specification" for all figures.

I hope I have sufficiently addressed all matters and look forward to hearing from you.

Yours sincerely

Peter Hayes

Peter Hayes

Digitally signed by Peter Hayes
Date: 2026.05.13 11:34:59 +01'00'
