# Supplementary material for: Development and qualification of an enzyme-linked immunosorbent assay to detect human serum immunoglobulin G reactive to multiple lineages of Lassa virus nucleoprotein
Source: PLoS One. 2026 Jul 2;21(7):e0340568. doi: 10.1371/journal.pone.0340568 (PMC13327249; doi:10.1371/journal.pone.0340568)
Supplement: Attachment — Submitted filename: Review_LASV_ELISA.docx [file pone.0340568.s012.docx]

This manuscript is describing the creation of anti-LASV NP IgG ELISA that can be used to detect background exposures to LASV. Understanding background exposures to LASV is critically important to understand baseline incidence in vaccine trials. Given that the manufacturer who provided the assays for previous trials is no longer producing the ELISA plates, this study focuses on recreating a comparable assay to use going forward.

I appreciate the thoroughness of which the team optimized and tested all parameters of the ELISA. With respect to the presentation of the study, I do feel that the results section is bloated with extraneous details that take away from the key highlights of this study: that a lineage IV NP antigen can detect anti-LASV NP IgG from areas where lineage II and III is expected to dominate, the creation of a reference standard on par with international reference standards (t**hough this data needs to be shown side-by-side),** that hemolyzed or samples undergoing freeze-thaw cycles can still be measured accurately, and most importantly the assay performance results are nearly identical to the commercially manufactured product.

I recommend that the authors edit the manuscript to remove the testing related to minute operator details. These results showing the differences in assay optimization could be their own methods manuscript but, in my opinion, take away from this manuscript. In my line-by-line comments below I have suggested some wording and phrasing edits to help the manuscript flow more smoothly to the reader, as well as some general comments. The discussion section should also be fleshed out to provide more context as to where and the data presented. Likewise, the abstract also needs to be reworked to reflect an updated and streamlined study.

I look forward to reading a revised manuscript.

__________________________________________________________________________

Line 68: Zalgen Labs, LLC (can be referred to as Zalgen or Zalgen Labs after the first mentioned, as has been done elsewhere in the manuscript).

Would recommend combining lines 61-66 and 80-88 into one paragraph, then 67-73 in another paragraph to help the reader understand the importance of creating these new assays.

Lines 73-79: Instead of describing the assay as is done in the methods, why is an ELISA the assay of choice to detect LASV exposures as opposed to dried blood spots or other tests?

Line 112: Suggest moving the “16-20 hours at 4C” to after the statement describing how the antigen is solubilized the antigen in PBS.

Lines 116-118: “with this washing step…” can be removed here. The washing step can be referenced as needed in the subsequent paragraph, “as described above”.

Line 118-119: Suggest to simply say that blocking and subsequent steps were performed at 37C; remove “initially” since all the data are reported at 37C.

Line 130: What is the primary active ingredient in the stop solution? Is it methylsulfuric acid or something else? What is the concentration of the active ingredient?

Lines 132-134: Last sentence of this paragraph can be removed.

**Lines 149-155, and elsewhere: The method of how the ELISA was performed is consistent except for the variables to be tested (protein concentration, washing method etc). The extraneous information is redundant in the results section.**

Lines 158-160: This should be in the methods section, not results.

Line 179: 20/202 is the NIBSC control, correct*?* ***Do you have a side by side comparison of 20/202 and the new pool for the selected conditions (2ug/mL Lineage IV)? This would be important to show.***

Line 210: How is this different from 20/202?

Line 212: Add to methods.

Paragraph from lines 225-238: When looking at the Nigerian and Liberia/SL samples separately, there is no statistical difference between the II/III/IV plates and IV plates. Rather than emphasizing the “trending significance”, it’s actually a good thing that they are not statistically significant differences because that’s what shows IV is comparable to II/III/IV in detecting NP from different lineages.

Evaluation of ELISA incubation parameters, dilution linearity, manual vs automatic plate washing: These sections can be removed without impacting the message of the study. The initial assay establishment could also be removed, and just state in the methods that only 2ug/mL was used.

The freeze-thaw and hemolyzed samples are important in the context of real-world practice, would suggest keeping these two sections.

Line 473 and Table S5:This is great and I would include this as a primary table.

Lines 494-497: Provide citation data and reference where in the manuscript (which tables/figures) are being used to provide this evidence.

Lines 511-512: Will this reference material be shared or only used internally for the trial?

Discussion in general: How do your results compare with other human NP LASV ELISA assays? Have other studies shown differences in performance when samples of varying quality were used? More context of how this assay compares to other LASV assays, and the importance of being able to develop these assays in West Africa, should be added. Limitations of the study should also be described in the discussion.

Line 524: Simpson

Table S4: Are there a total of 7 NIBSC reference samples? Previously it was only referred to as having one reference sample (20/206). Please clarify in the methods.

Line 609: a commercial Zalgen ELISA kit

Abstract: Rewrite the second and third paragraphs of the abstract to reflect the updated contents of the manuscript, emphasizing that lineage IV assay detected samples from II/II regions as well as the lineage II/III/IV assay, the generation of the reference serum, and concordance with the commercial assay.
