# Supplementary material for: Development and qualification of an enzyme-linked immunosorbent assay to detect human serum immunoglobulin G reactive to multiple lineages of Lassa virus nucleoprotein
Source: PLoS One. 2026 Jul 2;21(7):e0340568. doi: 10.1371/journal.pone.0340568 (PMC13327249; doi:10.1371/journal.pone.0340568)
Supplement: Peer review history

# Peer review: Decision Letter 0

**DOI:** 10.1371/journal.pone.0340568.r001

**Authors:** Masahiro Kajihara

4 Apr 2026

PONE-D-25-67874

Development and qualification of an enzyme-linked immunosorbent assay to detect human serum immunoglobulin G reactive to multiple lineages of Lassa virus nucleoprotein

PLOS One

Dear Dr. Hayes,

Thank you for submitting your manuscript to PLOS ONE. After careful consideration, we feel that it has merit but does not fully meet PLOS ONE’s publication criteria as it currently stands. Therefore, we invite you to submit a revised version of the manuscript that addresses the points raised during the review process.

Please submit your revised manuscript by May 19 2026 11:59PM. If you will need more time than this to complete your revisions, please reply to this message or contact the journal office at plosone@plos.org. When you're ready to submit your revision, log on to https://www.editorialmanager.com/pone/ and select the 'Submissions Needing Revision' folder to locate your manuscript file.

Please include the following items when submitting your revised manuscript:

- A letter that responds to each point raised by the academic editor and reviewer(s). You should upload this letter as a separate file labeled 'Response to Reviewers'.
- A marked-up copy of your manuscript that highlights changes made to the original version. You should upload this as a separate file labeled 'Revised Manuscript with Track Changes'.
- An unmarked version of your revised paper without tracked changes. You should upload this as a separate file labeled 'Manuscript'.

If you would like to make changes to your financial disclosure, please include your updated statement in your cover letter. Guidelines for resubmitting your figure files are available below the reviewer comments at the end of this letter.

If applicable, we recommend that you deposit your laboratory protocols in protocols.io to enhance the reproducibility of your results. Protocols.io assigns your protocol its own identifier (DOI) so that it can be cited independently in the future. For instructions see: https://journals.plos.org/plosone/s/submission-guidelines#loc-laboratory-protocols. Additionally, PLOS ONE offers an option for publishing peer-reviewed Lab Protocol articles, which describe protocols hosted on protocols.io. Read more information on sharing protocols at https://plos.org/protocols?utm_medium=editorial-email&utm_source=authorletters&utm_campaign=protocols.

As the corresponding author, your ORCID iD is verified in the submission system and will appear in the published article. PLOS supports the use of ORCID, and we encourage all coauthors to register for an ORCID iD and use it as well. Please encourage your coauthors to verify their ORCID iD within the submission system before final acceptance, as unverified ORCID iDs will not appear in the published article. *Only*  the individual author can complete the verification step; PLOS staff *cannot*  verify ORCID iDs on behalf of authors.

We look forward to receiving your revised manuscript.

Kind regards,

Masahiro Kajihara, PhD

Academic Editor

PLOS One

Journal Requirements:

When submitting your revision, we need you to address these additional requirements.

1.Please ensure that your manuscript meets PLOS ONE's style requirements, including those for file naming. The PLOS ONE style templates can be found at

https://journals.plos.org/plosone/s/file?id=wjVg/PLOSOne_formatting_sample_main_body.pdf and

https://journals.plos.org/plosone/s/file?id=ba62/PLOSOne_formatting_sample_title_authors_affiliations.pdf

2. Please include a complete copy of PLOS’ questionnaire on inclusivity in global research in your revised manuscript. Our policy for research in this area aims to improve transparency in the reporting of research performed outside of researchers’ own country or community. The policy applies to researchers who have travelled to a different country to conduct research, research with Indigenous populations or their lands, and research on cultural artefacts. The questionnaire can also be requested at the journal’s discretion for any other submissions, even if these conditions are not met. Please find more information on the policy and a link to download a blank copy of the questionnaire here: https://journals.plos.org/plosone/s/best-practices-in-research-reporting. Please upload a completed version of your questionnaire as Supporting Information when you resubmit your manuscript.

3. Thank you for stating the following financial disclosure:

“This work was funded by Coalition for Epidemic Preparedness Innovations (CEPI) (award reference number: 276869) and the EDCTP2 programme supported by the European Union EDCTP (grant number: RIA2019LV-3053).”

Please state what role the funders took in the study.  If the funders had no role, please state: "The funders had no role in study design, data collection and analysis, decision to publish, or preparation of the manuscript."

If this statement is not correct you must amend it as needed.

Please include this amended Role of Funder statement in your cover letter; we will change the online submission form on your behalf.

4. Please note that funding information should not appear in any section or other areas of your manuscript. We will only publish funding information present in the Funding Statement section of the online submission form. Please remove any funding-related text from the manuscript.

5. We note that your Data Availability Statement is currently as follows: “All relevant data are within the manuscript and its Supporting Information files.”

Please confirm at this time whether or not your submission contains all raw data required to replicate the results of your study. Authors must share the “minimal data set” for their submission. PLOS defines the minimal data set to consist of the data required to replicate all study findings reported in the article, as well as related metadata and methods (https://journals.plos.org/plosone/s/data-availability#loc-minimal-data-set-definition).

For example, authors should submit the following data:

- The values behind the means, standard deviations and other measures reported;

- The values used to build graphs;

- The points extracted from images for analysis.

Authors do not need to submit their entire data set if only a portion of the data was used in the reported study.

If your submission does not contain these data, please either upload them as Supporting Information files or deposit them to a stable, public repository and provide us with the relevant URLs, DOIs, or accession numbers. For a list of recommended repositories, please see https://journals.plos.org/plosone/s/recommended-repositories.

If there are ethical or legal restrictions on sharing a de-identified data set, please explain them in detail (e.g., data contain potentially sensitive information, data are owned by a third-party organization, etc.) and who has imposed them (e.g., an ethics committee). Please also provide contact information for a data access committee, ethics committee, or other institutional body to which data requests may be sent. If data are owned by a third party, please indicate how others may request data access.

6. We notice that your supplementary figures are uploaded with the file type 'Figure'. Please amend the file type to 'Supporting Information'. Please ensure that each Supporting Information file has a legend listed in the manuscript after the references list.

7. Please include your full ethics statement in the ‘Methods’ section of your manuscript file. In your statement, please include the full name of the IRB or ethics committee who approved or waived your study, as well as whether or not you obtained informed written or verbal consent. If consent was waived for your study, please include this information in your statement as well.

8. If the reviewer comments include a recommendation to cite specific previously published works, please review and evaluate these publications to determine whether they are relevant and should be cited. There is no requirement to cite these works unless the editor has indicated otherwise.

[Note: HTML markup is below. Please do not edit.]

Reviewer's Responses to Questions

**Comments to the Author**

1. Is the manuscript technically sound, and do the data support the conclusions?

The manuscript must describe a technically sound piece of scientific research with data that supports the conclusions. Experiments must have been conducted rigorously, with appropriate controls, replication, and sample sizes. The conclusions must be drawn appropriately based on the data presented.

Reviewer #1: Yes

Reviewer #2: Yes

**********

2. Has the statistical analysis been performed appropriately and rigorously?

Reviewer #1: Yes

Reviewer #2: Yes

**********

3. Have the authors made all data underlying the findings in their manuscript fully available?

The PLOS Data policy requires authors to make all data underlying the findings described in their manuscript fully available without restriction, with rare exception (please refer to the Data Availability Statement in the manuscript PDF file). The data should be provided as part of the manuscript or its supporting information, or deposited to a public repository. For example, in addition to summary statistics, the data points behind means, medians and variance measures should be available. If there are restrictions on publicly sharing data—e.g. participant privacy or use of data from a third party—those must be specified.

Reviewer #1: Yes

Reviewer #2: Yes

**********

4. Is the manuscript presented in an intelligible fashion and written in standard English?

PLOS ONE does not copyedit accepted manuscripts, so the language in submitted articles must be clear, correct, and unambiguous. Any typographical or grammatical errors should be corrected at revision, so please note any specific errors here.

Reviewer #1: Yes

Reviewer #2: Yes

**********

5. Review Comments to the Author

Please use the space provided to explain your answers to the questions above. You may also include additional comments for the author, including concerns about dual publication, research ethics, or publication ethics. (Please upload your review as an attachment if it exceeds 20,000 characters)

Reviewer #1: This manuscript is describing the creation of anti-LASV NP IgG ELISA that can be used to detect background exposures to LASV. Understanding background exposures to LASV is critically important to understand baseline incidence in vaccine trials. Given that the manufacturer who provided the assays for previous trials is no longer producing the ELISA plates, this study focuses on recreating a comparable assay to use going forward.

I appreciate the thoroughness of which the team optimized and tested all parameters of the ELISA. With respect to the presentation of the study, I do feel that the results section is bloated with extraneous details that take away from the key highlights of this study: that a lineage IV NP antigen can detect anti-LASV NP IgG from areas where lineage II and III is expected to dominate, the creation of a reference standard on par with international reference standards (though this data needs to be shown side-by-side), that hemolyzed or samples undergoing freeze-thaw cycles can still be measured accurately, and most importantly the assay performance results are nearly identical to the commercially manufactured product.

I recommend that the authors edit the manuscript to remove the testing related to minute operator details. These results showing the differences in assay optimization could be their own methods manuscript but, in my opinion, take away from this manuscript. In my line-by-line comments below I have suggested some wording and phrasing edits to help the manuscript flow more smoothly to the reader, as well as some general comments. The discussion section should also be fleshed out to provide more context as to where and the data presented. Likewise, the abstract also needs to be reworked to reflect an updated and streamlined study.

I look forward to reading a revised manuscript.

__________________________________________________________________________

Line 68: Zalgen Labs, LLC (can be referred to as Zalgen or Zalgen Labs after the first mentioned, as has been done elsewhere in the manuscript).

Would recommend combining lines 61-66 and 80-88 into one paragraph, then 67-73 in another paragraph to help the reader understand the importance of creating these new assays.

Lines 73-79: Instead of describing the assay as is done in the methods, why is an ELISA the assay of choice to detect LASV exposures as opposed to dried blood spots or other tests?

Line 112: Suggest moving the “16-20 hours at 4C” to after the statement describing how the antigen is solubilized the antigen in PBS.

Lines 116-118: “with this washing step…” can be removed here. The washing step can be referenced as needed in the subsequent paragraph, “as described above”.

Line 118-119: Suggest to simply say that blocking and subsequent steps were performed at 37C; remove “initially” since all the data are reported at 37C.

Line 130: What is the primary active ingredient in the stop solution? Is it methylsulfuric acid or something else? What is the concentration of the active ingredient?

Lines 132-134: Last sentence of this paragraph can be removed.

Lines 149-155, and elsewhere: The method of how the ELISA was performed is consistent except for the variables to be tested (protein concentration, washing method etc). The extraneous information is redundant in the results section.

Lines 158-160: This should be in the methods section, not results.

Line 179: 20/202 is the NIBSC control, correct? Do you have a side by side comparison of 20/202 and the new pool for the selected conditions (2ug/mL Lineage IV)? This would be important to show.

Line 210: How is this different from 20/202?

Line 212: Add to methods.

Paragraph from lines 225-238: When looking at the Nigerian and Liberia/SL samples separately, there is no statistical difference between the II/III/IV plates and IV plates. Rather than emphasizing the “trending significance”, it’s actually a good thing that they are not statistically significant differences because that’s what shows IV is comparable to II/III/IV in detecting NP from different lineages.

Evaluation of ELISA incubation parameters, dilution linearity, manual vs automatic plate washing: These sections can be removed without impacting the message of the study. The initial assay establishment could also be removed, and just state in the methods that only 2ug/mL was used.

The freeze-thaw and hemolyzed samples are important in the context of real-world practice, would suggest keeping these two sections.

Line 473 and Table S5:This is great and I would include this as a primary table.

Lines 494-497: Provide citation data and reference where in the manuscript (which tables/figures) are being used to provide this evidence.

Lines 511-512: Will this reference material be shared or only used internally for the trial?

Discussion in general: How do your results compare with other human NP LASV ELISA assays? Have other studies shown differences in performance when samples of varying quality were used? More context of how this assay compares to other LASV assays, and the importance of being able to develop these assays in West Africa, should be added. Limitations of the study should also be described in the discussion.

Line 524: Simpson

Table S4: Are there a total of 7 NIBSC reference samples? Previously it was only referred to as having one reference sample (20/206). Please clarify in the methods.

Line 609: a commercial Zalgen ELISA kit

Abstract: Rewrite the second and third paragraphs of the abstract to reflect the updated contents of the manuscript, emphasizing that lineage IV assay detected samples from II/II regions as well as the lineage II/III/IV assay, the generation of the reference serum, and concordance with the commercial assay.

Reviewer #2: This manuscript describes the development and qualification of an ELISA for the detection of anti-LASV nucleoprotein (NP)-specific IgG antibodies in human serum. The study is generally well designed and addresses an important need for serological tools in Lassa fever epidemiology and vaccine studies. The use of WHO reference standards and the systematic evaluation of assay performance (precision, linearity, specificity, and dynamic range) are notable strengths.I consider this work suitable for publication after minor revision. The comments below are intended to improve clarity, transparency, and confidence in the assay validation.

1. The generation of the reference serum pool raises an important point that would benefit from further clarification and experimental support. While the samples were collected prior to vaccination in a GPC-based vaccine trial and are therefore presumed to reflect natural infection, it is not fully demonstrated that the observed reactivity is specific to LASV NP. In particular, it remains unclear whether the high ELISA signals used to select samples for pooling reflect true NP-specific antibody responses or could include non-specific or cross-reactive binding. Additional supporting evidence would strengthen this point, such as confirmation of NP-specific reactivity by Western blot, or demonstration that these samples also exhibit serological evidence of prior LASV exposure (for example, pre-existing GPC-specific antibodies). Clarifying these aspects would increase confidence in the validity of the reference standard used for assay calibration.

2. It would be helpful to clarify whether recombinant NP antigens from different production lots were evaluated during assay development. If such experiments were performed, including this information would strengthen the manuscript. If not, assessing lot-to-lot variability could further improve confidence in the robustness and reproducibility of the assay.

3. The use of mean + 3 standard deviations to define the assay cutoff is acceptable. However, the robustness of the cutoff could be further strengthened by complementary approaches such as ROC curve analysis using well-defined positive and negative samples (e.g., WHO reference panel and naïve controls), if feasible.

4. Line 43: The statement that LASV has “pandemic potential” may be overstated or requires appropriate supporting references. Please provide a citation or revise the wording.

5. Line 48: Please define “IAVI” at first mention.

6. The term “positivity point” is used throughout the manuscript. More commonly used terminology such as “cutoff” or “threshold” may improve clarity.

7. Line 509 – “LAV” should be corrected to “LASV”.

**********

6. PLOS authors have the option to publish the peer review history of their article (what does this mean?). If published, this will include your full peer review and any attached files.

If you choose “no”, your identity will remain anonymous but your review may still be made public.

**Do you want your identity to be public for this peer review?**  For information about this choice, including consent withdrawal, please see our Privacy Policy.

Reviewer #1: No

Reviewer #2: No

**********

[NOTE: If reviewer comments were submitted as an attachment file, they will be attached to this email and accessible via the submission site. Please log into your account, locate the manuscript record, and check for the action link "View Attachments". If this link does not appear, there are no attachment files.]

To ensure your figures meet our technical requirements, please review our figure guidelines: https://journals.plos.org/plosone/s/figures

You may also use PLOS’s free figure tool, NAAS, to help you prepare publication quality figures: https://journals.plos.org/plosone/s/figures#loc-tools-for-figure-preparation.

NAAS will assess whether your figures meet our technical requirements by comparing each figure against our figure specifications.

Attachment Submitted filename: PONE-D-25-67874.pdf

Attachment Submitted filename: Review_LASV_ELISA.docx

# Author comment: Author response to Decision Letter 1

**DOI:** 10.1371/journal.pone.0340568.r002

13 May 2026

I have responded to all reviewer and editor comments in the "response to reviewers" letter uploaded with this resubmission

Attachment Submitted filename: Response to reviewers.pdf

# Peer review: Decision Letter 1

**DOI:** 10.1371/journal.pone.0340568.r003

**Authors:** Masahiro Kajihara, Masahiro Kajihara

15 Jun 2026

Development and qualification of an enzyme-linked immunosorbent assay to detect human serum immunoglobulin G reactive to multiple lineages of Lassa virus nucleoprotein

PONE-D-25-67874R1

Dear Dr. Hayes,

We’re pleased to inform you that your manuscript has been judged scientifically suitable for publication and will be formally accepted for publication once it meets all outstanding technical requirements.

Within one week, you’ll receive an e-mail detailing the required amendments. When these have been addressed, you’ll receive a formal acceptance letter and your manuscript will be scheduled for publication.

An invoice will be generated when your article is formally accepted. Please note, if your institution has a publishing partnership with PLOS and your article meets the relevant criteria, all or part of your publication costs will be covered. Please make sure your user information is up-to-date by logging into Editorial Manager at Editorial Manager® and clicking the ‘Update My Information' link at the top of the page. For questions related to billing, please contact billing support.

If your institution or institutions have a press office, please notify them about your upcoming paper to help maximize its impact. If they’ll be preparing press materials, please inform our press team as soon as possible -- no later than 48 hours after receiving the formal acceptance. Your manuscript will remain under strict press embargo until 2 pm Eastern Time on the date of publication. For more information, please contact onepress@plos.org.

Kind regards,

Masahiro Kajihara, PhD

Academic Editor

PLOS One

Additional Editor Comments (optional):

Reviewers' comments:

Reviewer's Responses to Questions

**Comments to the Author**

1. If the authors have adequately addressed your comments raised in a previous round of review and you feel that this manuscript is now acceptable for publication, you may indicate that here to bypass the “Comments to the Author” section, enter your conflict of interest statement in the “Confidential to Editor” section, and submit your "Accept" recommendation.

Reviewer #1: All comments have been addressed

Reviewer #2: All comments have been addressed

**********

2. Is the manuscript technically sound, and do the data support the conclusions?

The manuscript must describe a technically sound piece of scientific research with data that supports the conclusions. Experiments must have been conducted rigorously, with appropriate controls, replication, and sample sizes. The conclusions must be drawn appropriately based on the data presented.

Reviewer #1: Yes

Reviewer #2: Yes

**********

3. Has the statistical analysis been performed appropriately and rigorously?

Reviewer #1: Yes

Reviewer #2: Yes

**********

4. Have the authors made all data underlying the findings in their manuscript fully available?

The PLOS Data policy requires authors to make all data underlying the findings described in their manuscript fully available without restriction, with rare exception (please refer to the Data Availability Statement in the manuscript PDF file). The data should be provided as part of the manuscript or its supporting information, or deposited to a public repository. For example, in addition to summary statistics, the data points behind means, medians and variance measures should be available. If there are restrictions on publicly sharing data—e.g. participant privacy or use of data from a third party—those must be specified.

Reviewer #1: Yes

Reviewer #2: Yes

**********

5. Is the manuscript presented in an intelligible fashion and written in standard English?

PLOS ONE does not copyedit accepted manuscripts, so the language in submitted articles must be clear, correct, and unambiguous. Any typographical or grammatical errors should be corrected at revision, so please note any specific errors here.

Reviewer #1: Yes

Reviewer #2: Yes

**********

6. Review Comments to the Author

Please use the space provided to explain your answers to the questions above. You may also include additional comments for the author, including concerns about dual publication, research ethics, or publication ethics. (Please upload your review as an attachment if it exceeds 20,000 characters)

Reviewer #1: Great work with the revised manuscript. The comments have been sufficiently addressed. This is a now a compelling, cohesive story of a robust multi-lineage LASV NP IgG ELISA and new clinical references.

One very minor comment -- line 478 should be "Discussion" not "Conclusions".

Reviewer #2: (No Response)

**********

7. PLOS authors have the option to publish the peer review history of their article (what does this mean?). If published, this will include your full peer review and any attached files.

If you choose “no”, your identity will remain anonymous but your review may still be made public.

**Do you want your identity to be public for this peer review?**  For information about this choice, including consent withdrawal, please see our Privacy Policy.

Reviewer #1: No

Reviewer #2: No

**********

# Editor report: Acceptance letter

**DOI:** 10.1371/journal.pone.0340568.r004

**Authors:** Masahiro Kajihara, Masahiro Kajihara

PONE-D-25-67874R1

PLOS One

Dear Dr. Hayes,

I'm pleased to inform you that your manuscript has been deemed suitable for publication in PLOS One. Congratulations! Your manuscript is now being handed over to our production team.

At this stage, our production department will prepare your paper for publication. This includes ensuring the following:

* All references, tables, and figures are properly cited

* All relevant supporting information is included in the manuscript submission,

* There are no issues that prevent the paper from being properly typeset

You will receive further instructions from the production team, including instructions on how to review your proof when it is ready. Please keep in mind that we are working through a large volume of accepted articles, so please give us a few days to review your paper and let you know the next and final steps.

Lastly, if your institution or institutions have a press office, please let them know about your upcoming paper now to help maximize its impact. If they'll be preparing press materials, please inform our press team within the next 48 hours. Your manuscript will remain under strict press embargo until 2 pm Eastern Time on the date of publication. For more information, please contact onepress@plos.org.

You will receive an invoice from PLOS for your publication fee after your manuscript has reached the completed accept phase. If you receive an email requesting payment before acceptance or for any other service, this may be a phishing scheme. Learn how to identify phishing emails and protect your accounts at https://explore.plos.org/phishing.

If we can help with anything else, please email us at customercare@plos.org.

Thank you for submitting your work to PLOS ONE and supporting open access.

Kind regards,

PLOS ONE Editorial Office Staff

on behalf of

Dr. Masahiro Kajihara

Academic Editor

PLOS One
